# Chemotherapeutic efficacy of nano formulation capecitabine@SNPs and gamma radiation: a novel metronomic approach for breast cancer treatment

**DOI:** 10.1007/s13346-026-02099-5

**Published:** 2026-05-15

**Authors:** Marwa I. Ghanim, Ibrahim Y. Abdelrahman, Ayda K. Kelany, Mostafa A. Askar, Mustafa Gharib, Heba K. Abdelhakim

**Affiliations:** 1 Biochemistry Division, Chemistry Department, Faculty of Science, Cairo University, Giza, Egypt; 2 Radiation Biology Department, National Center for Radiation Research and Technology, Egyptian Atomic Energy Authority, Cairo, Egypt; 3 Department of Genomic Medicine, Cairo University Hospitals, Cairo University, Cairo, 11599 Egypt; 4 Applied Science Research Center, Applied Science Private University, Amman, 11831 Jordan

**Keywords:** Breast Cancer, Capecitabine, Nano formulation, Radiotherapy, Metronomic Strategy

## Abstract

Despite the prevalence of various infectious and pandemic diseases worldwide, cancer remains the leading cause of death. Clinical approaches for treating cancer vary depending on the tumor type and aggressiveness, including surgery, chemo-, radio-, immune- and hormone therapies. However, the efficacy of these treatments is hindered by the high mortality rate during chemotherapy and radiotherapy, primarily due to severe side effects.This experimental study was conducted on 40 female Sprague-Dawley (SD) rats (n=8). A metronomic treatment strategy was employed using intraperitoneal administration of a small dose of capecitabine loaded onto silica nanoparticles (Cap@SNPs) (377mg/kg) once weekly for a month, either singly or in combination with a low dose of gamma ionizing radiation (IR) (0.5 Gy/5 days) over six sessions, against a 7,12-dimethylbenz[a]anthracene (DMBA) induced mammary carcinoma model in SD rats. The antitumor effect of Cap@SNPs and/or γ-radiation was assessed by tumor volume measurements, survival analysis, biochemical and haematological parameters and gene expression profiling of selected apoptotic and anti-apoptotic genes. Cell cycle phase distribution, apoptotic cells (Annexin V) and the expression of some immunomodulatory proteins were analyzed by flow cytometry. Histopathological examination was performed using hematoxylin and eosin (H&E) staining. The expression of estrogen receptor (ER), progesterone receptor (PR) and human epidermal growth factor receptor 2 (HER2/neu) proteins in mammary tissue was assessed by immunohistochemistry (IHC). The initial tumor volume across all groups was 32.8 ± 1.8 mm^3^, reaching 86 ± 1.9 mm^3^ in the positive control at Day 30 but only 5.3 ± 0.5 mm^3^ in the Cap@SNPs + IR group. The death rate decreased from 75% in the positive control to 12.5% in the combined-treated group. Gene expression analysis revealed the downregulation and upregulation of the anti-apoptotic gene (*Bcl2*) and the apoptotic genes (*Bax*, *Fas* and *Caspase-8*), respectively, in the combination therapy. Flow cytometric analysis showed cell cycle arrest at the G1 phase and increased early apoptosis in the combined-treated group. The findings were verified by histopathological examination and immunohistochemical analysis of mammary tumor tissues. In conclusion, administration of a small capecitabine dose via silica nanoparticle delivery, along with fractionated radiotherapy, achieved substantial tumor regression, improved survival and effective cell-cycle arrest in mammary tumor-bearing rats. These findings support the integration of metronomic chemotherapy with nanoscale drug delivery systems and fractionated radiotherapy as a promising multimodal strategy to enhance anti-tumor efficacy, reduce dose-related toxicity and ultimately improve therapeutic outcomes.

**Graphical abstract:**

**Figure:**
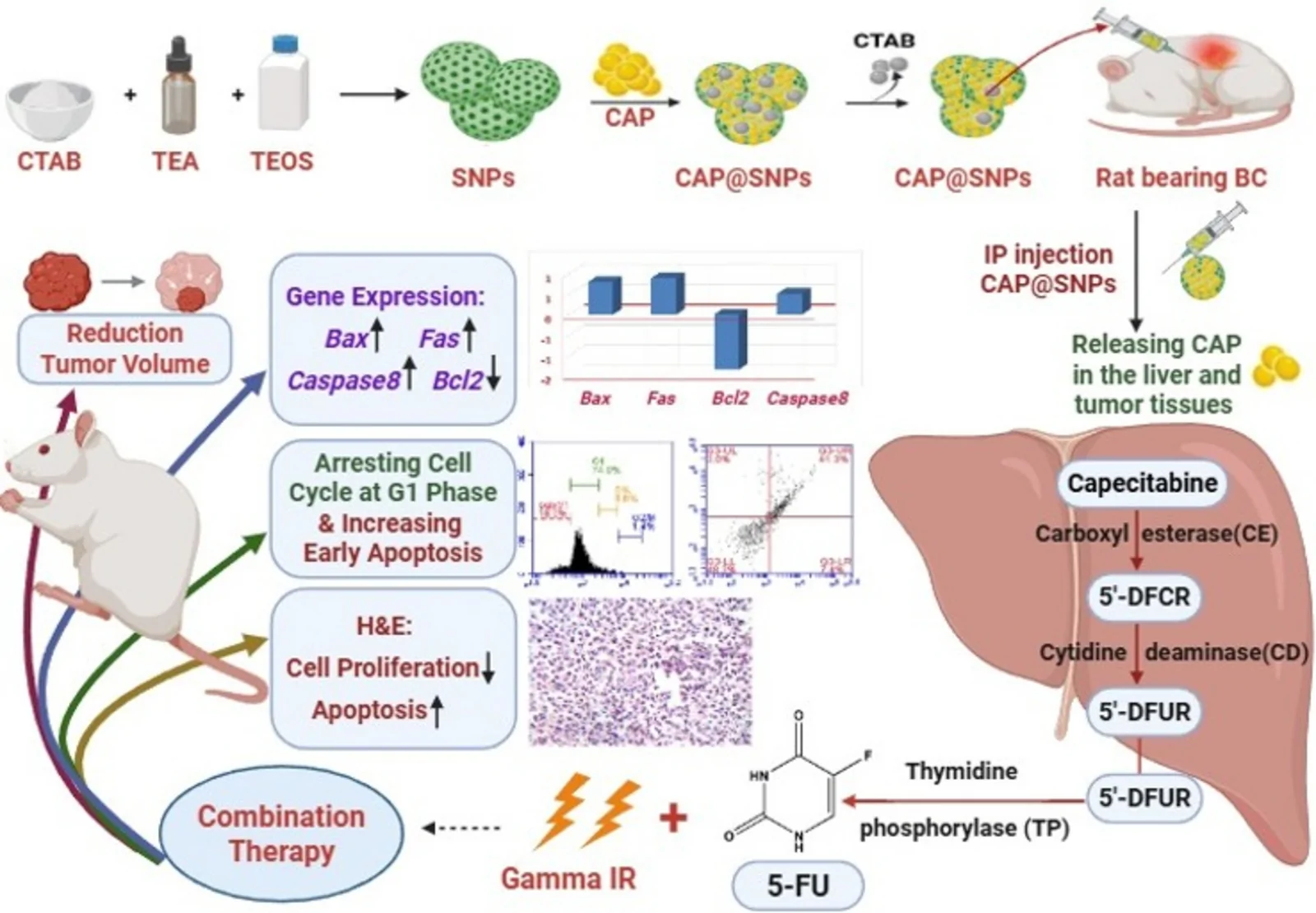
Schematic representation of the synthesis, metabolism and synergistic antitumor efficacy of capecitabine-loaded silica nanoparticles (CAP@SNPs) combined with gamma irradiation. Mesoporous silica nanoparticles (SNPs) were synthesized using CTAB, TEA and TEOS and subsequently loaded with capecitabine (CAP). Following intraperitoneal (IP) injection into a breast cancer (BC) rat model, CAP is enzymatically converted in the liver and tumor tissues into the active chemotherapeutic agent, 5-fluorouracil (5-FU). The combination of the released 5-FU and localized gamma irradiation (Gamma IR) significantly reduces tumor volume. This therapeutic synergy is mediated by the induction of cell cycle arrest at the G1 phase, increased early apoptosis, favorable modulation of apoptotic gene expression (upregulation of Bax, Fas, Caspase-8 and downregulation of Bcl2) and confirmed by histopathological (H&E) assessment showing decreased cell proliferation.

## Introduction

Cancer is a major global health threat, alongside the prevalence of infectious and pandemic diseases [1]. Breast cancer (BC) presents one of the most common malignancies, challenging women’s health, with an increased mortality rate. In 2020, it represented (17.7–19) % of all new cancer diagnoses globally [2]. Regarding breast cancer, the estimated incidence of new breast cancer cases was around 2.26 million in 2020. It is the fifth leading cause of cancer mortality worldwide [3]**,** with 685,000 deaths, a cumulative of 685,000 breast cancer-related deaths worldwide in that year [4]. According to statistics from the World Health Organization (WHO), approximately 2.3 million women were diagnosed with breast cancer and 670, 000 deaths occurred globally (WHO, 2022). Kim et al. determined that, on average, 1 in 20 women globally will receive a breast cancer diagnosis during their lifetime. If current trends persist, by 2050, there will be 3.2 million new breast cancer cases and a million breast cancer-related fatalities annually, with the increase disproportionately impacting nations with a low Human Development Index (HDI) [5].

Cancer incidence and mortality rates have continued to rise dramatically over the past several decades. Despite substantial global efforts to improve prevention, diagnosis and treatment, cancer remains one of the most globally widespread diseases; its expanding burden reflects persistent shortcomings in current therapeutic strategies. Treatment strategies vary based on tumor type, stage, molecular characteristics, and aggressiveness. These approaches encompass chemotherapy, radiotherapy, immunotherapy, hormonal therapy and surgery. Conventional cancer therapies like chemotherapy, surgery, and radiotherapy are associated with limited selectivity for malignant cells, causing damage to healthy tissues, leading to severe systemic toxicities, in addition to facing issues like drug resistance and poor tumor penetration. Even newer approaches, such as immunotherapy, while offering greater target specificity, may provoke immunogenic reactions and show restricted efficacy in solid tumors due to poor immune cell infiltration and an immunosuppressive tumor microenvironment. Consequently, combination regimens, such as chemoradiotherapy, have become standard practice in many malignancies to overcome resistance mechanisms and enhance tumor control [6]. Ongoing research is continually optimizing established therapies and developing new ones using nanostructures to reduce drug toxicity in healthy tissues and increase efficacy by targeting tumor angiogenesis.

Within this chemotherapeutic landscape, capecitabine, commercially known as Xeloda, is an orally administered fluoropyrimidine prodrug widely used in the treatment of breast, gastric, and colorectal cancers. It is presently sanctioned by the FDA, either as monotherapy or in combination regimens, such as with docetaxel for metastatic breast cancer. Following absorption, capecitabine undergoes a three-step enzymatic activation pathway, which involves the fluoropyrimidine carbamate metabolized in the liver and tumor by carboxyl esterases (CE) to 5′-deoxy-5-fluorocytidine (5′-DFCR), then by cytidine deaminase (CD) to 5′-deoxy-5-fluorouridine (5′-DFUR). Finally, conversion of (5'-DFUR) to 5-Fluorouracil (5-FU) by thymidine phosphorylase (TP), which occurs predominantly intratumorally, thereby achieving higher drug concentrations at the tumor site while reducing systemic exposure [7]. Its antineoplastic efficacy is primarily attributed to inhibition of thymidylate synthase, resulting in impaired DNA synthesis, cell-cycle arrest, and induction of apoptosis [8]. Despite these advantages, oral administration of capecitabine is associated with a spectrum of adverse effects, including gastrointestinal disturbances (nausea, vomiting, diarrhea, abdominal pain), fatigue, and dermatological reactions such as hand–foot syndrome. More severe complications may involve myelosuppression, cardiotoxicity (e.g., ischemia and cardiomyopathy), hypersensitivity reactions and coagulation abnormalities. The drug is contraindicated in patients with severe renal impairment and its use during pregnancy poses a substantial risk of teratogenicity [9]. Collectively, these side effects exemplify a larger issue represented by the limited therapeutic index of many chemotherapeutic drugs, which continues to contribute considerably to treatment-related morbidity and mortality. In response to these limitations, alternative dosing strategies have gained increasing attention, among which metronomic chemotherapy (MCT) has emerged as a particularly promising approach. In addition, switching from oral to intraperitoneal administration improves absorption efficiency and blood bioavailability, while sparing healthy tissues and mitigating gastrointestinal adverse effects commonly associated with oral administration.

Metronomic chemotherapy (MCT) involves the long-term administration of chemotherapeutic agents at substantially lower doses than those used in conventional dose regimens, while maintaining the same route and pharmaceutical formulation. This strategy aims to achieve anti-tumor efficacy by inhibiting tumor angiogenesis, modulating the immune response and reducing systemic toxicity [10, 11]. Although this strategy offers a more sustainable and effective treatment option, its conventional drug-delivery system may be associated with certain risks, such as poor permeability, lack of solubility of drug molecules, suboptimal efficacy and inadequate targeting, and thereby unintended damage to healthy tissues [12]. These considerations underscore the need for optimized delivery platforms that can maximize the benefits of low-dose chemotherapy while minimizing its limitations.

Nanotechnology applications are at the forefront of medical innovation and a new revolution in science, by transforming drug delivery, diagnostics and regenerative medicine. Synthesizing nanoparticles is a promising approach to addressing cancer-related disorders by improving drug bioavailability and delivering drugs to targeted areas with high efficiency. Such systems are particularly relevant for cytotoxic agents, where enhanced delivery efficiency can directly translate into improved therapeutic indices. Newly synthesized nanoparticles can be used alone due to their intrinsic properties or in combination with other biomolecules to target cancer cells [13] [14]. Mesoporous silica nanoparticles (MSNs), among the various nanoplatforms explored, serve multifunctional roles in medicine as diagnostic agents, targeted drug delivery systems, and therapeutic platforms for cancer, neurological disorders and regenerative applications due to their unique physicochemical characteristics [15]. Mesoporous silica nanoparticles (MSNs) exhibit an ordered internal pore network with large surface area and tunable pore sizes, facilitating high drug loading and protecting encapsulated agents from premature degradation, thereby enhancing bioavailability and therapeutic efficacy [16]. It possesses internal mesopores with diameters around (2–6 nm), large pore volume (0.6–1 cm^3^/g), high surface area (700–1000 m^2^/g), tunable size approximately (50–200 nm) and abundant silanol (Si–OH) groups, which enable targeted functionalization with ligands, polymers or stimuli-responsive moieties to achieve controlled, site-specific drug release [17] [18]. The intrinsic chemical stability and favorable biocompatibility of silica support their use in physiological environments, where their mesoporous architecture allows the incorporation of both hydrophilic and hydrophobic drugs while maintaining structural integrity [19]. Moreover, tailoring particle size and pore structure enhances cellular uptake and can be optimized for passive or active targeting in cancer therapy, improving accumulation at diseased tissues while reducing systemic side effects [20]. Furthermore, stimuli-responsive modifications of MSNs, including pH-, redox- or enzyme-triggered systems, provide sophisticated control over drug release kinetics, enabling the design of smart nanocarriers for precision medicine applications [21]. The advantageous features of MSNs make them ideal platforms for designing multifunctional nano-systems.

As chemotherapeutic treatments advance and systemic anti-tumor therapies evolve, it is increasingly evident that single-modality treatment is insufficient to achieve optimal therapeutic efficacy. Radiation therapy remains one of the most effective and noninvasive treatment modalities [22]. There are two types of radiation, ionizing and non-ionizing. Gamma radiation (γ-rays) is a type of ionizing radiation. Low-dose radiation (LDR) is defined as ionizing radiation exposures below thresholds where deterministic effects occur but may still elicit biological responses, typically within the range of about 0.1 Gy up to approximately 2 Gy. This differs from conventional high-dose radiotherapy by emphasizing biological modulation rather than outright cytotoxicity [23]. Gamma rays exert ionizing effects by transferring certain amounts of energy to the irradiated cells, generating reactive oxygen species (ROS), producing cellular DNA damage and thereby inducing cell death or tumor stromal modulation, resulting from exposure to a low dose of radiation [24]. Furthermore, LDRT can be administered to primary and metastatic sites with minimal toxicity; it shows strong effectiveness by boosting the body's immune response against tumors and increasing tumor susceptibility to systemic therapies. These characteristics make LDRT demonstrate significant biological efficacy when integrated with chemotherapy, immunotherapy, hormone therapies, and surgery [23] [25]. In this context, ionizing gamma radiation can be delivered at low doses of radiotherapy (LDRT) as a compelling adjunct to multimodal cancer management.

In this study, we aim to evaluate the integrated multimodal strategy of using a newly synthesized nanoformulation of capecitabine encapsulated in mesoporous silica nanoparticles as a drug delivery system, administered intraperitoneally rather than conventionally orally, via customized metronomic dosing and in combination with a low dose of gamma radiation, against DMBA-induced mammary cancer in rat models.

## Materials and methods

### Chemicals

Capecitabine (Xeloda, 500 mg tablet form) is a chemotherapy medication that was purchased from the pharmacy as a product of the Genentech company. Tetraethyl orthosilicate (TEOS) (ADVENT CHEMBIO PVT.LTD, an ISO certificated company). Tri-ethanolamine (TEA) (SDFCL company, SD Fine-Chem Limited). Cetyltrimethylammonium bromide (CTAB), 98%, (ADVENT CHEMBIO PVT.LTD, an ISO certificated company) Merck Co. DMBA was obtained from Sigma Chemicals.

### Synthesis of mesoporous silica nanoparticles (SNPs)

Mesoporous silica nanoparticles (SNPs) were synthesized following the synthesis protocol outlined by Pan et al. with slight modifications [26]. Initially, 10 g of cetyltrimethylammonium chloride (CTAC) and 0.2 g of triethylamine (TEA) were dissolved in 100 mL of deionized (DI) water and subjected to vigorous stirring at 95 °C in a silicone oil bath for one hour. Subsequently, 7.5 mL of tetraethyl orthosilicate (TEOS) was added dropwise at a rate of 1 mL per minute into the dispersion. The resulting mixture was stirred for an additional hour under the same conditions (95 °C with vigorous stirring). Upon completion, the mixture was allowed to cool, and the SNPs were separated as pellets via centrifugation at 20,000 × g for 15 min. The collected particles underwent multiple washes with ethanol before being dried overnight at 60 °C in an oven. Following drying, the pellets were crushed and subjected to calcination in a muffle furnace (Thermo Scientific, Australia) at 550 °C for 7 h to ensure complete removal of the surfactant.

### Synthesis of capecitabine loading on mesoporous silica nanoparticles

In a glass vial, 100 mg of capecitabine were dissolved in 2 ml of ethanol. Subsequently, 100 mg of mesoporous silica nanoparticles (SNPs) were introduced into the solution, and the vial cap was securely sealed. The mixture was then incubated at room temperature with continuous agitation at 200 rotations per minute for 48 h. Following the incubation period, the resultant loaded nanoparticles were purified by centrifugation and were then dried at 40 °C, after which they were collected and kept for further work.

### Characterization of capecitabine-loaded mesoporous silica nanoparticles

The nanoparticles were characterized using various analytical techniques. Transmission electron microscopy (TEM) (JEM 1230 electron microscope with an accelerating voltage of 100 kV, JEOL, Tokyo, Japan), Dynamic Light Scattering (DLS) employing a (Zetasizer Nano ZN instrument from Malvern Panalytical Ltd, United Kingdom), and Fourier transform infrared (FTIR) spectroscopy (Bruker Alpha FTIR Spectrometer, NAWAH Co.) were utilized for comprehensive characterization of the nanoparticles. These techniques were employed to investigate nanoparticle size, morphology, surface charge, and optical properties. For TEM analysis, multiple independent micrographs (approximately ten images) were acquired from different regions of the same nanoparticle sample. Nanoparticle size distributions were obtained by measuring individual particle diameters using ImageJ software and the reported values represent the mean ± standard deviation calculated from these measurements. This approach is commonly used to ensure statistically meaningful size analysis in electron microscopy. Dynamic light scattering (DLS) and zeta potential measurements were performed in triplicate on the same nanoparticle batch under identical conditions. Fourier-transform infrared (FTIR) spectroscopy was performed once per sample, as FTIR is a qualitative and compositional technique with high reproducibility for chemical fingerprinting. A single representative spectrum was therefore considered sufficient to confirm the presence of characteristic functional groups and successful drug loading.

### Irradiation facilities

Irradiation was performed using a (^137^Cs γ-ray irradiator) type (Gamma-cell-40 Exactor) located at the National Center for Radiation Research and Technology (NCRRT), Nasr City, Egyptian Atomic Energy Authority (EAEA), Cairo, Egypt. The rats that underwent irradiation singly or combined with chemotherapy were irradiated with gamma rays (γ-rays) with a total dose of 3 Gy, fractioned into six sessions (0.5 Gy every 5 days), at a dose rate of (0.33 Gy/min), as illustrated in Table 1.

**Table 1:** The fractionated doses of gamma rays through the month of treatment

Day 0	Day 5	Day 10	Day 15	Day 20	Day 25	Day 30
Dose 1 (0.5Gy)	Dose 2 (0.5Gy)	Dose 3 (0.5Gy)	Dose 4 (0.5Gy)	Dose 5 (0.5Gy)	Dose 6 (0.5Gy)	Termination

### Experimental animals

Female Sprague-Dawley (SD) rats were obtained from the breeding unit at the NCRRT, EAEA, Cairo, Egypt.

The animals were housed in plastic cages in a well-ventilated room under standardized animal house conditions (12 h light and 12 h dark). They were housed in 10 open-topped polypropylene cages (45 × 30 × 18 cm), with 4 per cage, under standard laboratory conditions, including all hygienic measures, ventilation, temperature (24 ± 2 °C), relative humidity (50 ± 10%), and a 12-h light/dark cycle. Rats were fed a growing, fattening feed (21% protein) and water ad-libitum throughout the experimental period. Experimental rats were permitted to acclimate to laboratory conditions for one week before the experiment.

The study was approved by the research ethics committee for experimental studies (Human and Animal subjects) at NCRRT, EAEA, Cairo, Egypt, following the 3Rs principle for animal experimentation (Replace, Reduce and Refine). It is organized and operated according to the Council for International Organizations of Medical Sciences (CIOMS) and the International Council for Laboratory Animal Science (ICLAS) International Guiding Principles for Biomedical Research Involving Animals, 2012 (serial number: F/50A/23).

### Experimental design and treatment protocol

This experimental study was conducted on 40 female Sprague-Dawley (SD) rats. Eight rats served as negative controls. The remaining 32 rats were used as induced mammary tumor-bearing models. Mammary carcinoma was induced via subcutaneous injection of 7,12-dimethylbenz[a]anthracene (DMBA), dissolved in dimethyl sulfoxide (DMSO). DMBA was administered into the mammary glands at a dose of 25 mg per rat, following Rajendran et al. [27], with two doses: the first at zero time and the second after two weeks. The rats were weighed weekly and palpated to record the number, location and size of tumors. Tumor-bearing rats were classified based on tumor volume. At the onset of the treatment protocol, the average tumor volume across mammary tumor-bearing rats was 32.8 ± 1.8 mm³. The rats weighed approximately 220 ± 5 grams at the start of the treatment, with an age of 20 ± 0.5 weeks. The experimental design and treatment plan were as follows.

- I.Negative Control (n=8): served as normal rats; received distilled water, used as the dissolving agent in the nano-formulation drug during preparation. The remaining 32 rats, acting as mammary tumor-bearing models (tumor volume across the rats was 32.8 ± 1.8 mm³), were divided randomly into the following groups:
- II.Positive Control (n=8): represented untreated mammary tumor-bearing rats and received distilled water.
- III.IR (n=8): represented mammary tumor-bearing rats and treated via exposure to gamma irradiation (IR) by an accumulated dose of 3 Gy, fractionated into 6 sessions (0.5 Gy every 5 days) for one month.
- IV.Cap@SNPs (n=8): represented mammary tumor-bearing rats and treated with capecitabine loaded onto mesoporous silica nanoparticles (Cap@SNPs), injected intraperitoneally (IP) with 50% of the certified dose of capecitabine (377 mg/kg rat) as a metronomic dose once weekly for one month, where the approved dose of capecitabine is 755 mg per kg rat [[28].
- V. Cap@SNPs+IR (n=8): represented mammary tumor-bearing rats, treated with Cap@SNPs (377 mg/kg rat once weekly) and exposed to gamma irradiation (IR) (0.5 Gy every 5 days) as a combination therapy for one month.

### Tumor volume measurement

Tumor volumes in the positive control and different treated groups were measured for monitoring tumor progression across the month of treatment. It was measured by determining tumor dimensions using a Vernier Caliper every 4 days from the start of treatment until the end of the experiment. Tumor volumes were calculated using the following equation:

$$\mathrm V=\left(\mathrm W^2\times\mathrm L\right)/2$$

where V is the tumor volume (mm^3^), W is the tumor width (mm) (minor tumor axis) and L is the tumor length (mm) (major tumor axis) [29]. The treatment protocol started after the tumor mass reached 32.8 ± 1.8 mm³, with no substantial difference in tumor masses between groups at the initiation of treatment (p-value ˃ 0.05).

### Survival analysis

All cases under the current study were observed and the death cases were recorded. At the end of the present experiment, the final data were analyzed using the Kaplan–Meier equation analysis by SPSS, utilizing its functions for PLOT SURVIVAL HAZARD LOGSURV.

#### Survival and tumor inhibition metrics: MST, ILS%, TIR% and T/C%

All groups were monitored daily to check the mortality. The mean survival time (MST), percentage increase in life span (ILS%), tumor-growth inhibition rate percentage (TIR%) and the percentage of MST for treated animals versus the positive control (T/C%) were calculated.

The mean survival time (*MST*) was calculated according to the equation: MST = (day of first death + day of last death)/2. The percentage increase in life span (*ILS%*) was calculated using the equation (ILS% = (T − C)/C × 100), where T represents the MST of the treated animal, and C represents the MST of the positive control. Tumor-growth inhibition rate percentage (*TIR%*) = (C-T)/C × 100, where T represents the mean tumor volume of the treated animal, and C represents the mean tumor volume of the positive control. (*T/C%*) (treated vs. control) was calculated as the MST of the treated animal/MST of the positive control × 100.

The National Cancer Institute (NCI) stipulates that an ILS% greater than 25% and a T/C% over 125% signify substantial anticancer activity of the medication [30] [31].

#### Euthanization and collection of samples

At the end of the experiment, rats from all groups were sacrificed under gentle anesthesia with intraperitoneal (IP) thiopental (50mg/kg) and atropine (50µgm/kg) [74]. Blood samples from rats were obtained via heart puncture for determination of hematological parameters (Hb, RBCs, HCT, WBCs, lymphocytes and platelets) and biochemical parameters such as liver functions (GOT and GPT), kidney functions (urea and creatinine), inflammatory marker (C-reactive protein; CRP) and tumor markers (cancer antigen 125; CA125 and alpha-fetoprotein; AFP).

Then, the bodies of rats were dissected to collect mammary tissues from healthy control rats and mammary tumor tissues from the other groups for evaluation of oxidative stress marker; malondialdehyde (MDA), antioxidant markers; superoxide dismutase (SOD) and catalase (CAT); in addition, gene expression (Bax, Fas, Bcl2 and Caspase-8) determinations, flow cytometric analysis (cell cycle, Annexin V, PD1, p53, CD4, CD8, TGFβ, Bcl-2, FOXP3 and CD25), histopathological examination and immunohistochemical studies; estrogen receptor (ER), progesterone receptor (PR) and human epidermal growth factor receptor 2 (HER2/neu). Furthermore, liver and kidney tissues were collected for histopathological examination.

#### Biochemical and hematological assays 

Colorimetric determination of alanine aminotransferase (ALT) or aspartate aminotransferase (AST) was estimated according to the method of Reitman and Frankel [32]. Serum urea was estimated as described by Patton and Crouch [33], while serum creatinine was determined by buffered kinetic Jaffe’s reaction method as described by Bowers and Wong [34]. C-Reactive protein activity was assayed by the method [35], Alpha fetoprotein [36] and CA-125 [37]. The oxidative status was evaluated via assessment of superoxide dismutase (SOD), which was assayed using the method described by Nishikimi et al. [38]. Catalase (CAT) activity was assayed using the method described by [35, 36], and lipid peroxidation malondialdehyde (MDA) was measured according to the method described by [39]. Hematological parameters were demonstrated using a hematology analyzer (Sysmex XP300, Germany).

#### Gene expression analysis

The mRNA levels of *Bcl-2*, *Bax*, *Fas* Ligand, and *Caspase-8* were determined by real-time PCR. Total RNA was extracted from tissue samples from four groups using the procedure of ABT Total RNA Mini Extraction Kit (Spin Column), Catalogue number: ABT002. RNA concentration and purity were checked using a NanoDrop 2000 (Thermo Fisher Scientific). cDNA was synthesized from 500 ng RNA according to automated reverse transcription of the total RNA to generate complementary DNA (cDNA), ABT H-minus cDNA synthesis Kit, Catalogue number: ABT009. Real-time PCR was performed using Quanti Tect SYBR Green PCR Master Mix (Qiagen) together with specific primers. The following primer sequences were used: ***Bcl2*** fw 5′- GGATTGTGGCCTTCTTTGAGTTC-3′ and rev 5′-AGAGCGATGTTGTCCACCAG-3′; ***Bax*** fw 5′- ACAGGGTTTCATCCAGGATCGAG-3′ and rev 5′-AGCTCCATGTTGTTGTCCAGTTC-3′; ***Fas*** Ligand fw 5′- ACCTATGACGGGCACCAAAA-3′ and rev 5′-GCGGAAGACTGAGGAAATGA-3′; ***Caspase-8*** fw 5′- GGGGATGGCTACTGTGAAAA-3′ and rev 5′-CCTCGGGTTGTCTTTATTGC-3′; ***GAPDH*** (Reference) fw 5′- TGCCACTCAGAAGACTGTGG-3′ and rev 5′- TTCAGCTCTGGGATGACCTT −3′. Target gene expression levels were normalized to the *GAPDH* gene. All tests were performed independently in triplicate. The change in mRNA expression was measured using the delta–delta cycle-threshold (2 ^−^ ^Δ^^Δ^^C^^T^) method. The *Bax* to *Bcl-2* ratio was calculated to assess the cell's susceptibility to apoptosis [40]. 

#### Flow cytometric analysis

Isolated tissues for all groups were processed immediately. Mammary tissues were cut into pieces of 3 to 4 cm thickness, rinsed, and cleaned with PBS. Fresh tissues were mechanically dispersed, sequentially using 100 μm and 35 μm nylon cell-strainers (mesh) (BD-Falcon: 0877119). Specimens were pressed through the 100 μm mesh, which was placed in a tissue culture dish containing PBS/EDTA. The mesh was rinsed several times with cold PBS/EDTA at 4 °C and then centrifuged to form a cell suspension (310 g, 4 °C, 6 min). The supernatant was removed, and 5 mL of cold ethanol (80%, −20°C) was added dropwise to the fixed cells under constant, gentle vortexing. Before staining, the cells were kept at −20°C overnight. For cell cycle analysis, cells were treated with an appropriate volume of staining solution containing propidium iodide (PI) (50 μg/mL), PBS (30 μg/mL), and RNase-free DNase (0.3 mg/mL). For the apoptosis analysis, 100 µL of cell suspension was transferred into a 5 mL test tube. Then 5 µL of annexin-V kit reagent (cat. no. 556547, BD Pharmingen FITC Apoptosis Kit) was added. After that, 5 µL of PI (PE label) was added and the tubes were incubated in the dark at room temperature for 15 min. After incubation, the cells were resuspended in 200 µl 1X binding buffer. For the cell surface markers (p53, PD-1, CD4, CD8, Bcl-2, FOXP3, TGF-β, and CD25), cells were stained with 10 µL of monoclonal antibody in the dark for 30 min at room temperature. (BD-Pharmingen™, Cat. No. (557,026, 568,260, 554,836, 554,973, 568,664, 560,408, 563,143, 753,852, respectively). The cell cycle, apoptosis, and cell surface marker data were analyzed using BD Accuri-C6-Plus software (Biosciences, CA, USA) [24]. The following equation calculated the percentage of change:

$$\%\;\mathrm{of}\;\mathrm{change}=\left(Treatedmean-Controlmean\right)/Controlmean\times100$$

#### Histopathological and immunohistochemical examination

The rats were dissected to collect liver and kidney tissues, in addition to the breast tissue from the negative control and breast cancer masses from the other groups. The tissues were fixed in 10% formalin saline for 24 h, washed in tap water and followed by dehydration using serial dilutions of alcohol (methyl, ethyl and absolute ethyl). Samples were embedded in paraffin after being cleared in xylene and at 56 °C in a hot air oven for 24 h. Using a slide microtome, paraffin-embedded tissue blocks were prepared for sectioning at 4 μm thickness. The obtained sections were collected on glass slides, deparaffinized and stained using hematoxylin and eosin (H&E) for routine light electronic microscope examination [41].

Additional sections were prepared for immunohistochemical (IHC) staining and for recording the immunoreactive score (IRS) of HER2/neu, ER and PR on mammary tissues from all groups. The sections were stained by Estrogen Receptor alpha Monoclonal Antibody (SP1), cat no. PA5-85,171, Progesterone Receptor Monoclonal Antibody (Alpha PR6), cat no: MA1-411, and ErbB2 (HER-2) Polyclonal Antibody, cat no: PA5-115,352 (Invitrogen, Thermo Scientific, USA), at a dilution of 1:200. The images were captured by a LABOMED Fluorescence microscope LX400, cat no: 9126000; US and LABOMED camera software, USA.

The evaluation of immune-staining was performed and the immune-positive cells were counted in each region of interest (ROI) using a counting grid and their proportion among the total counterstained cell population was determined. The stained areas of the ROI were digitally marked and the percentage of stained areas was determined using a computer program.

The protein expression intensity was calculated using the immunoreactive score (IRS) for IHC data interpretation. The immune-reactive score (IRS) gives a range of (0–12) as a product of multiplication between the positive cells proportion score (0–4) and staining intensity score (0–3), as shown in Table 2.

**Table 2:** The immune-reactive score (IRS)

A (percentage of positive cells)	B (intensity of staining)	IRS score (multiplication of A and B)
0 = no positive cells	0 = no color reaction	0–1 = negative
1 = < 10% of positive cells	1 = mild reaction	2–3 = mild
2 = 11–50% positive cells	2 = moderate reaction	4–8 = moderate
3 = 51–80% positive cells	3 = intense reaction	9–12 = strongly positive
4 = > 80% positive cells	Final IRS score (A × B): 0–12	

#### Statistical analysis

Data were presented as the Mean ± SEM (Standard Error of the Mean). Statistical analyses were conducted using IBM SPSS Statistics for Windows, Version 22.0 (IBM, Armonk, NY, USA). Differences among multiple groups were evaluated using One-way Analysis of Variance (One-way ANOVA), followed by Fisher’s least significant difference (LSD) post hoc test for pairwise comparisons between group means. Survival analyses were conducted using the Kaplan–Meier method. The statistical significance was indicated by a (P-value ≤ 0.05), high significance by a (P-value ≤ 0.01) and non-significance by a (P-value > 0.05).

## Results

### Characterization of SNPs and capecitabine-loaded SNPs

**Fig. 1:**
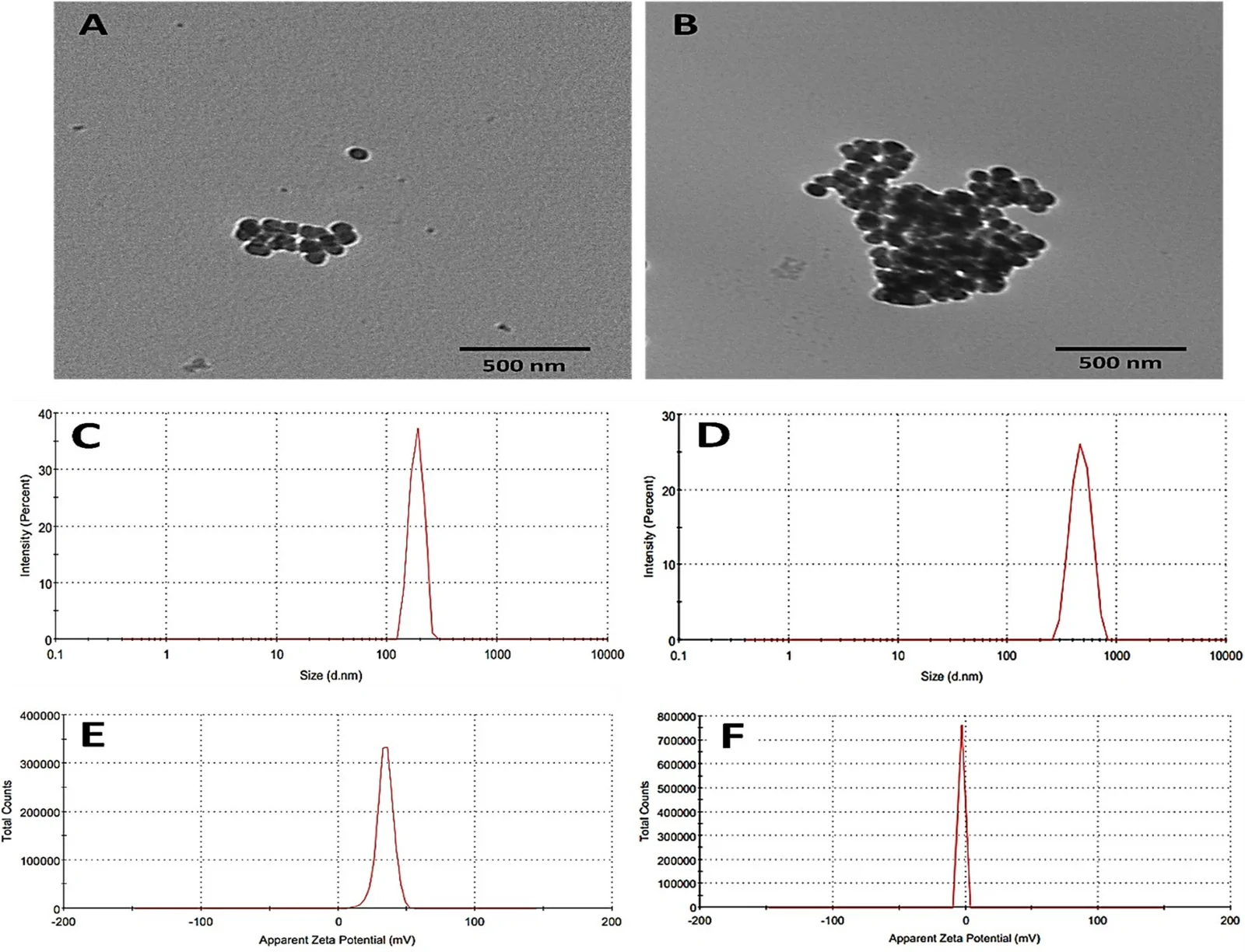
Characterization of Capecitabine-loaded SNPs. TEM images of SNPs (**A**) and (**B**) capecitabine-loaded SNPs. Hydrodynamic diameter (Dh) of SNPs **(C)** and capecitabine-loaded SNPs (**D**). Zeta potential of SNPs (**E**) and Capecitabine-loaded SNPs (**F**)

The size and shape of SNPs and capecitabine-loaded SNPs were characterized using TEM. As depicted in Fig. 1A and B, both SNPs and capecitabine-loaded SNPs exhibited primarily spherical shapes, with approximate average diameters of 60 ± 3 nm and 65 ± 2 nm, respectively. Furthermore, dynamic light scattering (DLS) analysis was employed to determine the hydrodynamic diameter (Dh) of the NPs. The results, illustrated in Fig. 1C and D, indicated that the Dh of mesopores SNPs was 185.7 nm, while capecitabine-loaded SNPs exhibited a Dh value of approximately 474.2 nm. The observed increase in hydrodynamic diameter of the capecitabine-loaded SNPs may be attributed to minimal NP aggregation, as evidenced by TEM imaging (Fig. 1B).

The notable increase in hydrodynamic diameter following capecitabine loading can be attributed primarily to changes in surface chemistry and colloidal behavior rather than an increase in the solid core size. This interpretation is supported by the significant shift in zeta potential from + 34.2 mV for bare SNPs to − 2.83 mV for capecitabine-loaded SNPs (Fig. 1E and F). The reduction in surface charge weakens electrostatic repulsion between nanoparticles, favoring transient clustering or soft agglomeration in suspension. Such reversible associations are commonly observed in mesoporous silica nanoparticles after drug loading, particularly when surface charges are partially shielded by adsorbed drug molecules.

Our findings revealed a significant change in the Zeta potential values between SNPs and capecitabine-loaded SNPs. The Zeta potential of SNPs was determined to be around +34.2 mV, while capecitabine-loaded SNPs exhibited a Zeta potential value of −2.83 mV (Fig. 1E and F). This obvious change in the Zeta potential value of capecitabine-loaded SNPs may be due to the abundant negatively charged OH groups that adsorbed capecitabine on the surface of SNPs.

**Fig. 2:**
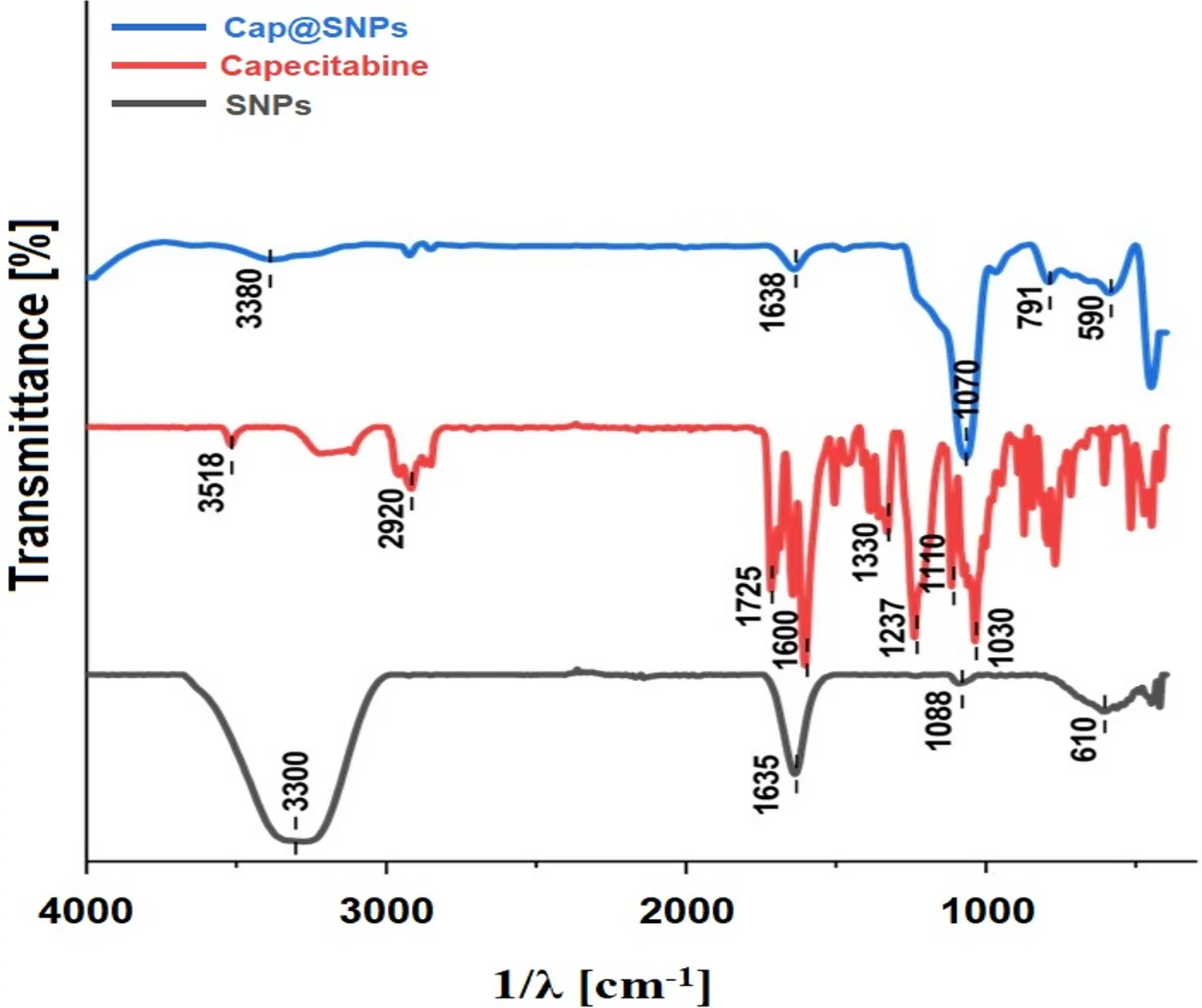
FT-IR spectra of SNPs, Capecitabine, and Capecitabine-loaded SNPs

Fourier-transform infrared spectroscopy was used to investigate the chemical structure of the silica nanoparticles, capecitabine and the capecitabine-loaded silica nanoparticles and to confirm the successful loading of capecitabine into SNPs (Fig. 2).

The FTIR analysis revealed the characteristic vibrational features of SNPs. A weak absorption band at ~1088 cm^−1^ [42] corresponded to the asymmetric stretching of Si-O-Si linkages, confirming the formation of the siloxane network. A broad peak centered around 3300 cm^−1^ [43] was assigned to O-H stretching vibrations from surface silanol groups and physisorbed water, while a strong band at ~1635 cm^−1^ [43] was attributed to H-O-H bending of adsorbed water molecules. In addition, a weaker absorption peak was observed near 610 cm-1 [43], which could be associated with low-frequency Si-O-Si vibrations (Fig. 2).

The FTIR spectrum of capecitabine exhibited a band at 1030 cm^−1^, assigned to C-N stretching, while the peak at 1110 cm^−1^ corresponds to C-O-C stretching. The absorption at 1237 cm^−1^ is attributed to C-O stretching, and the band at 1330 cm^−1^ arises from the C-F bond. The peak at 1600 cm^−1^ is assigned to C=N stretching, and the strong band at 1725 cm^−1^ corresponds to the carbonyl C=O stretching of the carbamate group. The signal at 2920 cm^−1^ is due to C-H stretching of aliphatic groups, and the broad band at 3518 cm^−1^ is assigned to O-H stretching of hydrogen-bonded hydroxyl groups [44] (Fig. 2).

On the other hand, the FTIR spectrum of Capecitabine-loaded silica nanoparticles retained the characteristic Si-O-Si vibrations of the silica network, including the asymmetric stretching at ~1070 cm^−1^, the broad O-H stretching around 3380 cm^−1^, and H-O-H bending at ~1635 cm^−1^, indicating that the silica framework remained intact (Fig. 2). Compared with the spectrum of free capecitabine, several characteristic capecitabine peaks were masked or disappeared in the loaded nanoparticles. These spectral changes suggest effective encapsulation of capecitabine within the pores of the silica nanoparticles.

### Tumor volume

**Fig. 3:**
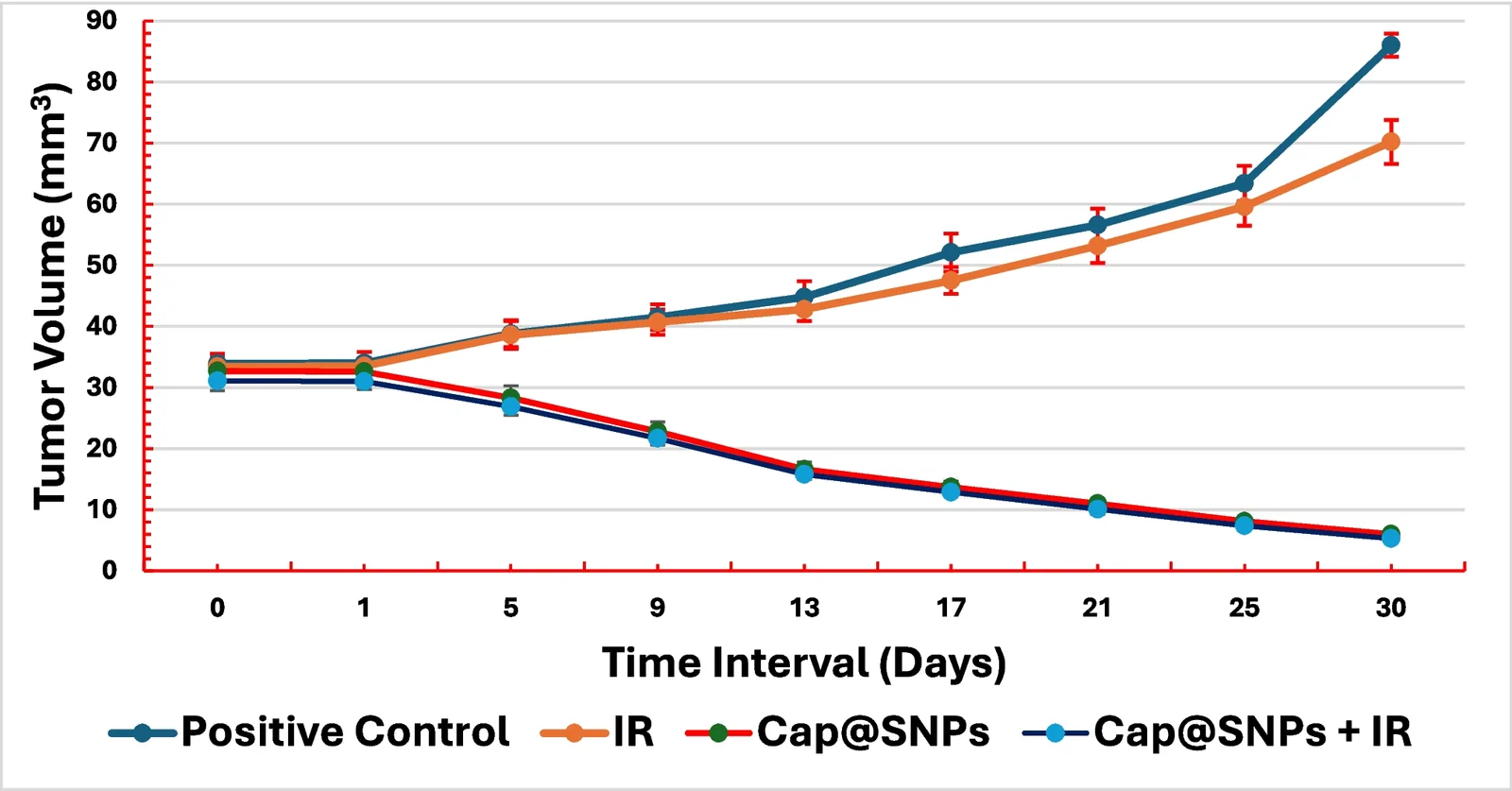
Changes in the tumor volume (mm^3^) in the positive control and treated groups through 30 days of treatment

The initial tumor volume (mm^3^) across all groups was 32.8 ± 1.8 mm^3^. Throughout the month of treatment, the untreated-induced breast cancer rats (positive control) group showed continuous increases, whereas the singly or combined treated groups (IR, Cap@SNPs and Cap@SNPs + IR), show decreases. Day 30 revealed an increase in tumor volume to 86 ± 1.9 mm^3^ in the positive control; furthermore, a decrease in the IR group to 70.2 ± 3.6 and marked regression in the Cap@SNPs & Cap@SNPs + IR groups to 6 ± 0.5 and 5.3 ± 0.5, respectively, as indicated in Table 3. Changes in tumor volume (mm^3^) over 30 days of treatment across the different treatment groups are presented in Fig. 3.

**Table 3:** Tumor volume (mm^3^) in the positive control and treated groups through 30 days of treatment

Groups	Positive Control	Treated Groups		
Time (Days)		IR	Cap@SNPs	Cap@SNPs + IR
Day 0	33.9 ± 1.6	33.4 ± 1.7	32.7 ± 2.3	31.1 ± 1.6
Day 1	34.0 ± 1.8	33.5 ± 1.3	32.6 ± 2.1	31.0 ± 1.3
Day 5	38.8 ± 2.2	38.6 ± 2.3	28.3 ± 2^a* b*^	26.9 ± 1.4^a* b*^
Day 9	41.5 ± 2.1	40.7 ± 2.1	22.8 ± 1.6^a* b*^	21.7 ± 1.1^a* b*^
Day 13	44.8 ± 2.6	42.8 ± 1.9	16.6 ± 1.2^a* b*^	15.8 ± 0.8^a* b*^
Day 17	52.1 ± 3.1	47.5 ± 2.2	13.7 ± 1^a* b*^	12.9 ± 0.6^a* b*^
Day 21	56.6 ± 2.7	53.2 ± 2.8	11 ± 0.8^a* b*^	10.1 ± 0.8^a* b*^
Day 25	63.4 ± 2.9	59.6 ± 3.1	8.1 ± 0.6^a* b*^	7.4 ± 0.7^a* b*^
Day 30	86 ± 1.9	70.2 ± 3.6^a^	6 ± 0.5^a* b*^	5.3 ± 0.5 ^a* b*^

(a, a*) indicate a significant and highly significant difference between the compared group and positive control group at *p* < 0.05 and *p* < 0.01, respectively, (b, b*) indicate a significant and highly significant difference between the compared group and gamma-irradiated group at *p* < 0.05 and *p* < 0.01, respectively

### Survival analysis

**Fig. 4:**
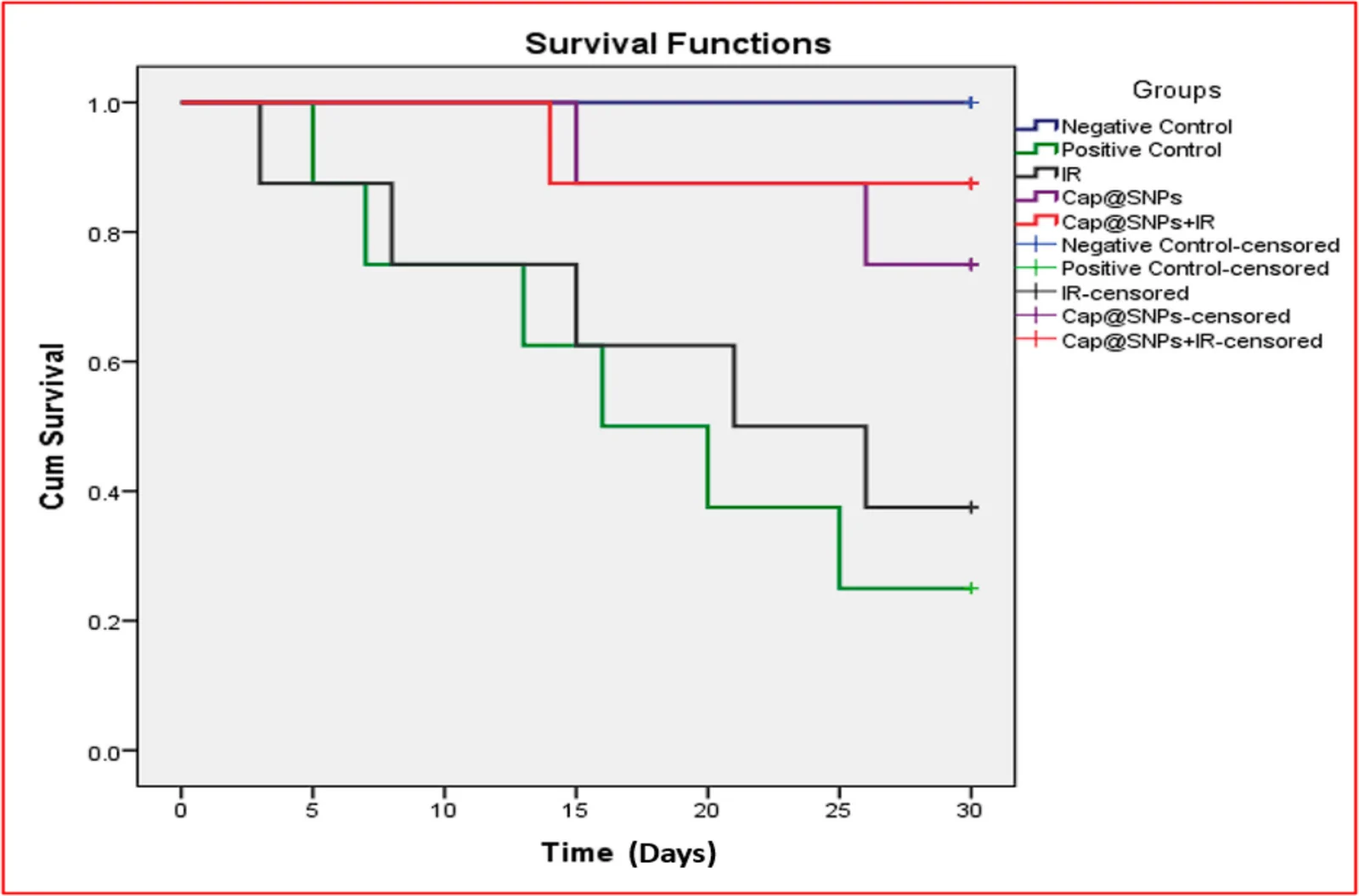
Kaplan–Meier survival curves of negative control, positive control and treated groups

The survival rate of the combined-treated (Cap@SNPs + IR) group increased significantly to 87.5%, compared with 25% in tumor-bearing rats (positive control). It exhibits 75% and 37.5% in the single-treated groups, Cap@SNPs and IR, respectively. The death rate reached 25% in the Cap@SNPs group and 62.5% in the IR group. Besides, a considerable decrease from 75% in the positive control group to 12.5% in the combined-treated (Cap@SNPs + IR) group, as indicated in Table 4 and Fig. 4.

**Table 4:** Statistical comparison of survival among negative control, positive control and treated groups

Parameters	Total cases	Events	Censored		
Groups		No. of death cases	Death rate	No. of cases still alive	Survival rate
Negative Control	8	0	0.0%	8	100.0%
Positive Control	8	6	75.0%	2	25.0%
IR	8	5	62.5%	3	37.5%
Cap@SNPs	8	2	25.0%	6	75.0%
Cap@SNPs + IR	8	1	12.5%	7	87.5%
Overall	40	14	35.0%	26	65.0%

Events: The no. of death cases and Censored: The no. of the observed cases still alive to the end of the experiment

### Survival and tumor inhibition metrics: MST, ILS%, TIR% and T/C%

Survival and tumor inhibition metrics, MST, ILS%, TIR%, and T/C%, in the positive control group and either single/or combined-treatment strategies, are calculated and presented in Table 5, where the single treatment with gamma radiation (IR) shows ILS% = 13.33% and T/C% = 113.33%. Furthermore, the treatment with Cap@SNPs singly and in combination (Cap@SNPs + IR) shows ILS% of 40% and 73.33%, respectively, and T/C% of 140% and 173.33%, respectively.

**Table 5:** Assessment of survival and tumor inhibition metrics: MST, ILS%, TIR% and T/C% in positive control and treated groups

Parameters	**MST (**Mean Survival Time**)**			ILS%	(MTV) Mean Tumor Volume (mm^3^)	TIR%	T/C%	Status
Groups	1 st death day	Last death day	MST					
Positive Control	5	25	15	0%	86	0%	100%	
IR	9	25	17	13.33%	70.2	18.37%	113.33%	Non-significant anti-tumor activity vs. the +ve control
Cap@SNPs	17	25	21	40%	6	93.02%	140%	Significant anti-tumor activity vs. the +ve control
Cap@SNPs + IR	26	26	26	73.33%	5.3	93.84%	173.33%	

*MST* The mean survival time, *ILS%* The percentage increase in life span, *MTV* The mean tumor volume, *TIR%* The tumor-growth inhibition rate percentage, *T/C%* The MST of (treated group vs. positive control group) percentage

### Biochemical parameters

The levels of liver functions (GOT, GPT), kidney functions (urea, creatinine), inflammatory marker (CRP), inflammatory markers (CA125 and AFP), antioxidant enzymes (SOD and CAT) and the lipid peroxidation marker (MDA) are shown in Table 6.

**Table 6:** Effect of Cap@SNPs and/or IR on biochemical parameters

Groups	Negative Control	Positive Control	Treated Groups		
Parameters			IR	Cap@SNPs	Cap@SNPs + IR
GOT (U/L)	145.14 ± 3.13^a* b*^	215.74 ± 4.64^b*^	188.66 ± 4.06^a*c*^	174.16 ± 3.76^a* b* c*^	154.81 ± 3.34^a* b*^
GPT (U/L)	38.70 ± 0.84^a* b*^	91.90 ± 1.98^b*^	78.05 ± 1.90^a*c*^	72.57 ± 1.57^a* b* c*^	62.87 ± 1.35^a* b*^
Urea (mg/dL)	47.41 ± 1.02^a* b*^	86.09 ± 1.85^b*^	68.32 ± 1.73^a* c*^	53.20 ± 1.14^a* b* c*^	48.38 ± 1.04^a* b*^
Creatinine (mg/dL)	0.60 ± 0.01^a* b*^	2.04 ± 0.05^b*^	1.09 ± 0.02^a* c*^	0.85 ± 0.02^a* b* c*^	0.83 ± 0.02^a* b*^
CRP (mg/L)	6.48 ± 0.14^a* b*^	10.82 ± 0.23^b^	10.15 ± 0.22^a c*^	7.25 ± 0.15^a* b*^	7.05 ± 0.15^a* b*^
CA125 (U/mL)	2.49 ± 0.05^a* b*^	7.74 ± 0.17	7.44 ± 0.16 ^c*^	3.48 ± 0.08^a* b* c^	3.05 ± 0.06^a* b*^
AFP (ng/mL)	0.58 ± 0.01^a* b*^	2.99 ± 0.07^b*^	2.48 ± 0.05^a* c*^	0.86 ± 0.02^a* b*^	0.85 ± 0.02^a* b*^
SOD (U/g tissue)	64.83 ± 1.40^a* b*^	17.40 ± 0.37^b*^	30.93 ± 0.67^a* c*^	43.52 ± 0.93^a* b* c*^	50.31 ± 1.09^a* b*^
CAT (U/g tissue)	75.47 ± 1.63^a* b*^	25.13 ± 0.5^b*^	33.85 ± 0.72^a* c*^	53.20 ± 1.14^a* b* c*^	59.99 ± 1.29^a* b*^
MDA (nmol/g tissue)	5.30 ± 0.11^a* b*^	33.46 ± 0.7^b*^	25.12 ± 0.54^a* c*^	13.52 ± 0.30^a* b* c*^	11.61 ± 0.25^a* b*^

(a, a*) indicate a significant and highly significant difference between the compared group and positive control at *p* < 0.05 and *p* < 0.01, respectively, (b, b*) indicate a significant and highly significant difference between the compared group and IR group at *p* < 0.05 and *p* < 0.01, respectively, (c, c*) indicate a significant and highly significant difference between the compared group and Cap@SNPs + IR group at *p* < 0.05 and *p* < 0.01, respectively

Relating to the liver functions, our results revealed a highly significant decrease (*P* < 0.01) in liver enzymes (GOT, GPT) in the treated groups with gamma radiation, Cap@SNPs and Cap@SNPs + IR when compared with their positive control groups. There was a significant decrease (*P* < 0.05) in GOT and GPT levels in the combined treated group (Cap@SNPs + IR) compared with the single treatment groups (Cap@SNPs) and (IR).

Regarding kidney functions (urea, creatinine), there was a highly significant decline (*P* < 0.01) in their levels in IR or Cap@SNPs either singly or in combination, in comparison with their positive control groups. Moreover, there was an exceedingly significant reduction (*P* < 0.01) in urea and creatinine levels between the Cap@SNPs and the solely treated groups.

For the inflammatory marker (CRP), our results recorded that there was a highly significant decrease (*P* < 0.01) in CRP in the serum of rats treated with the Cap@SNPs singly or in combination and a significant reduction (*P* < 0.05) in rats treated with IR singly when compared with the positive control. At the same time, they still represent a highly significant decrease (*P* < 0.01) in rats treated with IR when compared with the combined therapy group.

Our results related ton serum tumor markers (CA125 and AFP) showed a highly significant decrease (*P* < 0.01) in CA125 levels in the Cap@SNPs and Cap@SNPs + IR groups compared with the positive control and a highly significant decline (*P* < 0.01) in AFP levels across all treatments, including IR or Cap@SNPs, either singly or in combination, compared with the positive control. In addition, the integrated Cap@SNPs + IR treatment resulted in a highly significant decrease (*P* < 0.01) in CA125 levels compared with both single-treated groups and in AFP levels compared with the γ-radiation-treated group.

On the other hand, the antioxidant enzyme levels (SOD and CAT) showed that there was a highly significant increase (*P* < 0.01) in the antioxidant enzyme levels measured in the tissues of rats across all treated groups, compared with the positive control, with the maximum significant elevation (*P* < 0.01) in the combined group treated with Cap@SNPs + IR compared with the single-treated groups.

Regarding the lipid peroxidation marker (MDA), our results indicated a gradual, highly significant decrease (*P* < 0.01) in the different treated groups, especially in the combined group, compared with the positive control group. In addition, the results emphasized a highly significant elevation (*P* < 0.01) in MDA levels in the combined group compared with the solely treated groups.

### Hematological parameters

The levels of red blood cells (RBCs), hemoglobin (Hb), hematocrit (HCT), white blood cells (WBCs), lymphocytes (LYM) and platelets (PLT) are shown in Table 7.

**Table 7:** Effect of Cap@SNPs and/or IR on hematological parameters

Groups	Negative Control	Positive Control	Treated Groups		
Parameters			IR	Cap@SNPs	Cap@SNPs + IR
RBCs (10⁶/µL)	6.49 ± 0.14^a*^	2.78 ± 0.05	3.16 ± 0.05^a b*^	5.75 ± 0.13^a*b*^	5.41 ± 0.11^a*b*^
Hb (g/dL)	12.43 ± 0.27^a*^	6.00 ± 0.13	8.20 ± 0.17^a*b*^	9.75 ± 0.22^a*b*^	9.95 ± 0.22^a*b*^
HCT (%)	37.50 ± 0.8^a*^	17.93 ± 0.38	18.56 ± 0.39^b*^	34.21 ± 0.74^a*b*^	35.89 ± 0.78^a*^
WBCs (10^3^/µL)	5.70 ± 0.12^a*^	90.66 ± 1.96	8.53 ± 0.19^a*b^	12.43 ± 0.27^a*b*^	9.05 ± 0.19^a*b^
LYM (10^3^/µL)	38.70 ± 0.84^a*^	74.49 ± 1.61	66.78 ± 1.44^a*b*^	55.14 ± 1.19^a*b*^	53.20 ± 1.14^a*b*^
PLT (10^3^/µL)	985.96 ± 21.27^a*^	290.00 ± 1.97	150.91 ± 3.25^a b*^	744.06 ± 16.07^a*b*^	677.31 ± 14.61^a*b*^

(a, a*) indicates a significant and highly significant difference between the compared group and the positive control at *p* < 0.05 and *p* < 0.01, respectively; (b, b*) indicate a significant and highly significant difference between the compared group and the negative control at *p* < 0.05 and *p* < 0.01, respectively

Regarding the red blood cells (RBCs) and hemoglobin (Hb) in the positive control, they decreased by half compared to the negative control. In addition, there was a highly significant increase (*P* < 0.01) in RBCs and Hb in the treated group by Cap@SNPs singly or combined with IR, but a considerable increase (*P* < 0.05) in the irradiated group when compared with the positive group.

Furthermore, the hematocrits (HCT) increased significantly (*P* < 0.05) in the treated groups, either singly or in combination with radiation. Still, there was no significant difference (*P* > 0.05) between the irradiated group and the positive control.

Focusing on the white blood cells (WBCs) and the lymphocytes (LYM) levels, our results showed that there was a highly significant elevation in the positive control groups when compared with the negative control groups at (*P* < 0.01) and a highly significant decrease (*P* < 0.01) in the three treated groups by Cap@SNPs, radiation and combination when compared with the positive group.

On the other hand, there was a highly significant decrease (*P* < 0.01) in the level of the platelets (PLT) in the positive control group and the gamma-irradiated group when compared with the negative control, but there was a highly significant elevation (*P* < 0.01) in Cap@SNPs and Cap@SNPs + IR (combined treated group) when compared with the positive control group, while in the gamma-irradiated group of rats, a significant decrease was recorded (*P* < 0.05) in the platelets levels when compared with the positive control group.

### Gene expression analysis

**Fig. 5:**
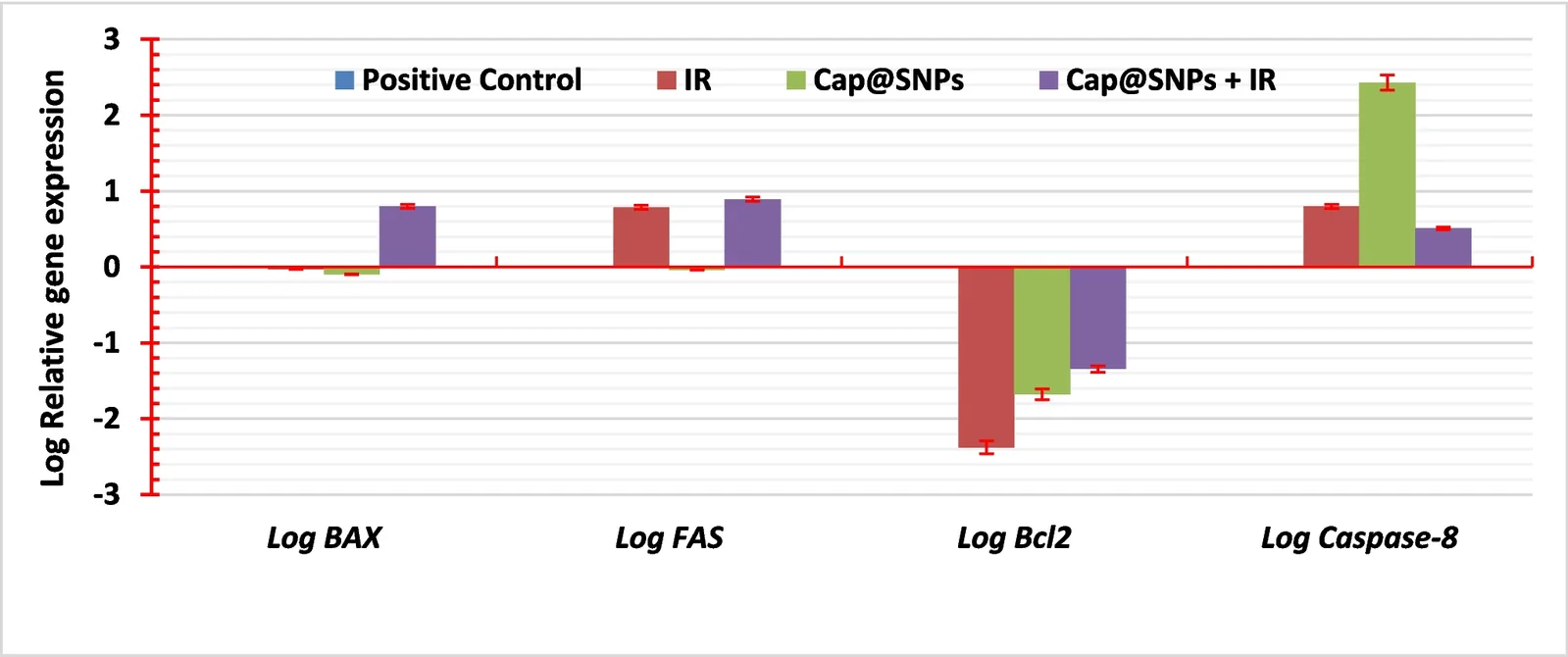
The log fold change in *Bax*, *Fas*, *Bcl2* and *Caspase-8* gene expression in positive control and treated groups

Gene expression analysis of the anti-apoptotic gene (Bcl2) and the pro-apoptotic genes (Bax, Fas and Caspase-8) across the positive control and the treated groups was measured. The results of the log fold change were presented in Table 8 and Fig. 5. Regarding the expression of the Bax gene, the combination therapy of Cap@SNPs and IR increased Bax expression by 80% (0.798 ± 0.026) compared to the positive control. In contrast, the individual treatments revealed negligible or slightly negative changes. The Cap@SNPs group showed a decrease by -10% (-0.095 ± 0.004) and IR by -3% (-0.031 ± 0.001) compared to the positive control. According to the Fas expression, it was significantly upregulated in the IR-treated group by 79% (0.786 ± 0.028) and further enhanced in the combination group by 89% (0.892 ± 0.029), but the Cap@SNPs-treated group showed a slight negative decrease by -4% (-0.040 ± 0.002) compared with the positive control. Focusing on Caspase-8 gene expression, our results showed a highly significant elevation in the combined-treated group by 51% (0.507 ± 0.017) and in the radiation group by 80% (0.798 ± 0.029), with a maximum significant increase in the Cap@SNPs-treated group by 243% (2.429 ± 0.101), compared to the positive group. On the other hand, the expression of the anti-apoptotic Bcl2 gene recorded a highly significant decrease in all treated groups, with the most profound suppression observed in the IR-treated group by -238% (-2.379 ± 0.085), followed by the Cap@SNPs group by -168% (-1.677 ± 0.070) and the combination group by -134% (-1.344 ± 0.044).

**Table 8:** The log fold change 2^−∆∆CT^ in *Bax*, *Fas*, *Bcl2* and *Caspase-8* gene expression in positive control and treated groups

Groups	Positive Control	Treated Groups		
Parameters		IR	Cap@SNPs	Cap@SNPs + IR
Log *Bax*	0.000 ± 0.000	−0.031 ± 0.001	−0.095 ± 0.004	0.798 ± 0.026
% Change	**0%**	**−3%**	**−10%**	**80%**
Log *Fas*	0.000 ± 0.000	0.786 ± 0.028	−0.040 ± 0.002	0.892 ± 0.029
% Change	**0%**	**79%**	**−4%**	**89%**
Log *Bcl2*	0.000 ± 0.000	−2.379 ± 0.085	−1.677 ± 0.070	−1.344 ± 0.044
% Change	**0%**	**−238%**	**−168%**	**−134%**
Log *Caspase-8*	0.000 ± 0.000	0.798 ± 0.029	2.429 ± 0.101	0.507 ± 0.017
% Change	**0%**	**80%**	**243%**	**51%**

The results indicated a discriminating apoptotic effect of the *Caspase-8* gene in the Cap@SNPs-treated group compared to other groups. Conversely, the gamma irradiation group exhibited a stronger anti-apoptotic effect through increased *Bcl-2* gene expression. Notably, the combination therapy group showed a significant increase in the apoptotic effect of the *Bax* gene. Additionally, the *Fas* gene exhibited enhanced apoptotic activity in both the Gamma irradiation and combination therapy groups compared to the Cap@SNPs-treated group.

The positive control group exhibits a *Bax*/*Bcl2* ratio of 0.0146. On the other side, gamma radiation (IR), capecitabine loaded onto silica nanoparticles (Cap@SNPs) and combination therapy (Cap@SNPs + IR) yielded ratios of 2.318 (157.7% increase), 1.059 (71.5% increase) and 3.673 (250.4% increase), respectively, as shown in Table 9.

**Table 9:** The Bax/Bcl2 Ratio and the cell status, ever resistance to the treatment or none

Parameters	*Bax*		*Bcl2*		*Bax*/*Bcl2* Ratio	% Change	Cell Status	
Groups	CT value	Conc. (ng/µl)	CT value	Conc. (ng/µl)			Resistance	Apoptosis
Positive Control	29.42	0.218	24.32	14.917	0.0146	0	**√**	
IR	31.90	0.037	34.71	0.016	2.318	157.7		**√**
Cap@SNPs	29.74	0.174	29.96	0.164	1.059	71.5		**√**
Cap@SNPs + IR	25.86	2.741	27.94	0.746	3.673	250.4		**√**

### Cap@SNPs and/or IR induced cell cycle arrest, apoptosis enhancement and immunomodulation in breast cancer-bearing rats

**Fig. 6:**
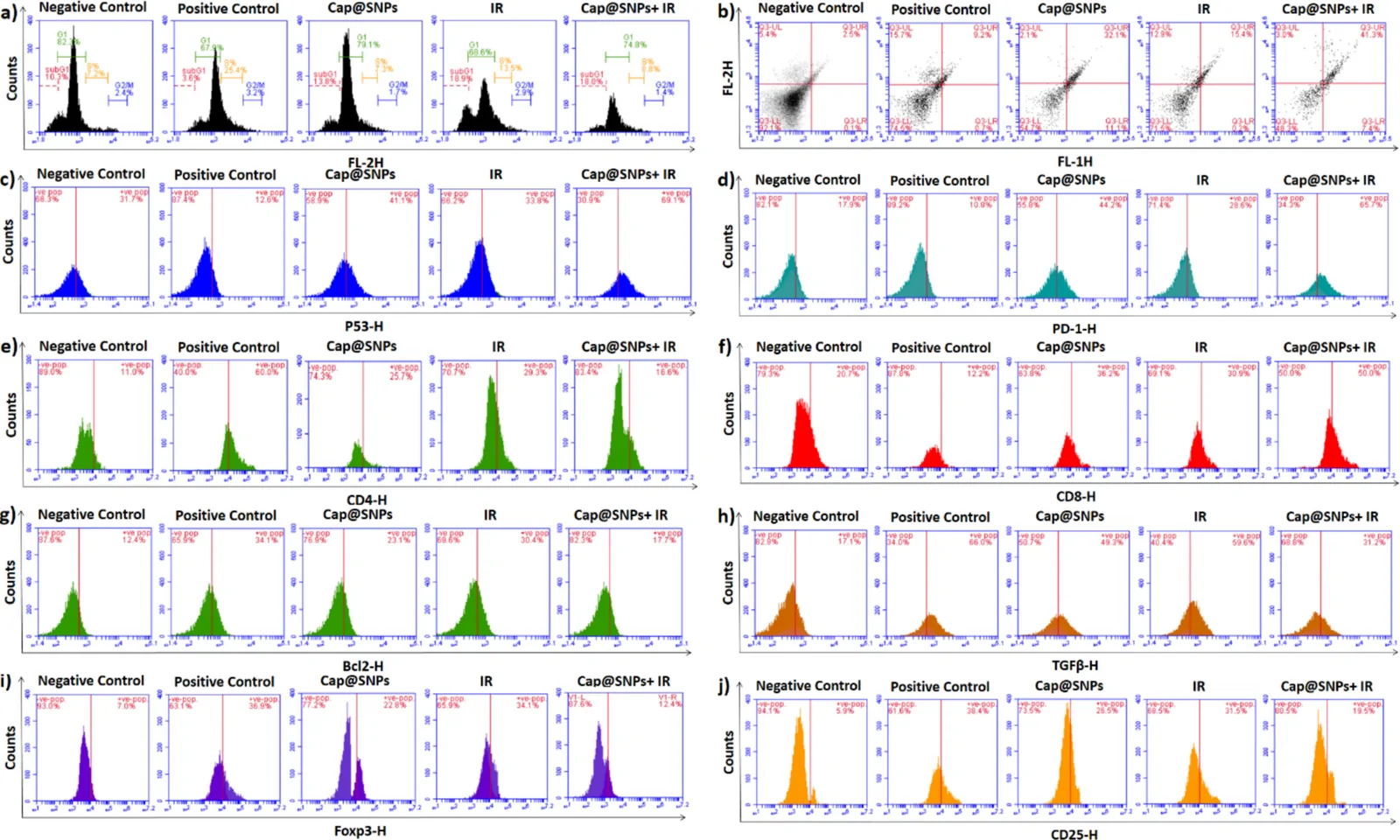
The influence of Cap@SNPs and/or IR on cell cycle, apoptosis, and immune-modulators in breast cancer-bearing rats. **a**) histogram of cell cycle; **b**) histogram of annexin-v; **c**) histogram of P53; **d**) histogram of PD-1; **e**) histogram of CD-4; **f**) histogram of CD-8; **g**) histogram of BCl-2; **h**) histogram of TGF-β; **i**) histogram of FOXP-3; **j**) histogram of CD-25

The present study examined the distribution of cell cycle phases in mammary tissues across all studied groups. The flow cytometry data analysis was illustrated in Table 10 and Fig. 6a. The distribution of cells was 10.3%, 82.8%, 7.2%, and 2.4% in the sub-G1 phase (DNA fragmentation), G1, S and G2/M phases of the negative control group, respectively. Significant deregulation of cell distribution was observed in the mammary tissue of the positive control group. Meanwhile, a considerable reduction of % change up to −65% and −57% occurred in the sub-G1 and G1 phases, respectively, noting that a significant increase in cell population of % change up to 292% and 1246% was seen in S and G2/M phases, respectively, compared to the -ve control. Regarding the effect of each of the single treatments on the cell distribution in different phases of the cell cycle, IR caused the majority of cells not to arrest at the sub-G1 and G1 phases and reached 0% and 93% respectively, this is comparable with 283% at sub-G1 and 125% at G1 phase induce arrest during the treatment with Cap@SNPs in comparison with + ve control group. By contrast, the distribution of cells in the S and G2/M phases was reduced by up to −10% and −90%, respectively, after IR treatment. Moreover, the pronounced decrease in cell population reached −74% and −95% in S and G2/M phases induced arrest after Cap@SNPs therapy compared to those of the + ve control.

**Table 10:** Cap@SNPs and/or IR induced cell cycle arrest in breast cancer in vivo

Groups	Negative Control	Positive Control	Treated Groups		
Parameters			IR	Cap@SNPs	Cap@SNPs + IR
Sub G1%	10.3	3.6	3.6	13.8	18
% Change	––-	**−65%**	**0%**	**283%**	**400%**
G1%	82.8	35.2	67.9	79.1	74.8
% Change	––-	**−57%**	**93%**	**125%**	**113%**
S %	7.2	28.2	25.4	7.3	8.8
% Change	––-	**292%**	**−10%**	**−74%**	**−69%**
G2/M %	2.4	32.3	3.2	1.7	1.4
% Change	––-	**1246%**	**−90%**	**−95%**	**−96%**

Regarding the combination therapy with IR and Cap@SNPs, there was an improvement in cell distribution within the tumor tissue of the treated rats. However, there was a 400% increase in apoptotic cells (sub-G1 phase), comparable to a 113% increase in the G1 phase and an induced arrest compared with the single IR treatment. On the contrary, significant reductions in cell population of −69% and −96% occurred in the S and G2/M phases, respectively, resulting in arrest compared to IR treatment alone. Annexin V-FITC staining was used to detect the tissue's apoptotic and necrotic rates. As shown in Table 11 and Fig. 6b, apoptosis in early stages was significantly increased in both groups treated with Cap@SNPs alone or in combination with IR when compared to the positive control group.

**Table 11:** Cap@SNPs and/or IR induced apoptosis in breast cancer in vivo

Groups	Negative Control	Positive Control	Treated Groups		
Parameters			IR	Cap@SNPs	Cap@SNPs + IR
Viable Cells	74.5	88.4	71.5	54.7	48.3
Necrotic Cells	15.7	7.8	12.9	2.1	3.0
Early Apoptosis	9.2	2.8	15.4	32.1	41.3
Late Apoptosis	0.7	0.2	0.2	11.1	7.4

Flow cytometry analysis of p53, PD-1, CD4, CD8, Bcl-2, TGF-β, FOXP3 and CD25 populations in mammary tissues in the different studied groups was summarized in Table 12.

**Table 12:** Cap@SNPs and/or IR modulate key proteins involved in regulating immunomodulation in breast cancer in vivo

Groups	Negative Control	Positive Control	Treated Groups		
Parameters			IR	Cap@SNPs	Cap@SNPs + IR
P53 (%)	32.30 ± 1.86^a*^	11.70 ± 0.64	31.30 ± 1.73^a*^	41.10 ± 1.73^a*b^	69.10 ± 3.62^a*b*^
PD-1 (%)	16.90 ± 0.97^a^	10.20 ± 0.56	29.40 ± 1.63^a*b*^	51.50 ± 2.16^a*b*^	70.60 ± 3.70^a*b*^
CD4 (%)	11.00 ± 0.63^a*^	60.00 ± 3.27	29.30 ± 1.62^a*b*^	25.70 ± 1.08^a*b*^	16.60 ± 0.87^a*^
CD8 (%)	20.70 ± 1.19^a*^	12.20 ± 0.66	30.90 ± 1.71^a*b*^	36.20 ± 1.52^a*b*^	50.00 ± 2.62^a*b*^
Bcl-2 (%)	12.40 ± 0.71^a*^	33.10 ± 1.8	30.40 ± 1.68^b*^	23.10 ± 0.97^a*b*^	17.70 ± 0.93^a*b^
TGF-β (%)	17.10 ± 0.98^a*^	66.00 ± 3.60	59.60 ± 3.30 ^b*^	49.30 ± 2.07^a*b*^	31.20 ± 1.63^a*b*^
FOXP3 (%)	7.00 ± 0.40^a*^	36.90 ± 2.01	34.10 ± 1.89^b*^	22.80 ± 0.96^a*b*^	12.40 ± 0.65^a*b^
CD25 (%)	5.90 ± 0.34^a*^	38.40 ± 2.09	31.50 ± 1.74^a*b*^	26.50 ± 1.11^a*b*^	19.50 ± 1.02^a*b*^

(a, a*) indicates a significant and highly significant difference between the compared group and the positive control group at *p* < 0.05 and *p* < 0.01, respectively, (b, b*) indicates a significant and highly significant difference between the compared group and the negative control group at *p* < 0.05 and *p* < 0.01, respectively

Regarding the p53 and PD-1 proteins, our results revealed a highly significant elevation in the proteins expression (*P* < 0.01), in rats treated by IR and Cap@SNPs alone or in combination, reaching (31.30 ± 1.73, 41.10 ± 1.73 and 69.10 ± 3.62), respectively in p53 and (29.40 ± 1.63, 51.50 ± 2.16 and 70.60 ± 3.70), respectively in PD-1 when compared with P53 and PD-1 in positive control (11.70 ± 0.64 and 10.20 ± 0.56), respectively.

For CD4 proteins, our results revealed that there was a highly significant decrease (*P* < 0.01) in CD4 expression level in Cap@SNPs, IR and combined treated groups, reaching (25.70 ± 1.08, 29.30 ± 1.62 and 16.60 ± 0.87), respectively, compared with the positive control group (60.00 ± 3.27). There is no significant difference in CD4 expression levels between the combined treated group and the untreated negative group.

On the other hand, showing a highly significant elevation (*P* < 0.01) in CD8 expression level in IR, Cap@SNPs and combined treated groups, reaching (30.90 ± 1.71, 36.20 ± 1.52 and 50.00 ± 2.62), respectively, compared with the positive control group (12.20 ± 0.66).

Focusing on the level of Bcl-2 and TGF-β expression, there is a highly significant decrease in the protein expression (*P* < 0.01), in rats treated by Cap@SNPs singly or combined, reaching (23.10 ± 0.97 and 17.70 ± 0.93), respectively, for Bcl-2 and (49.30 ± 2.07 and 31.20 ± 1.63), respectively, for TGF-β, when compared with Bcl-2 and TGF-β in the positive controls (33.10 ± 1.8 and 66.00 ± 3.60), respectively. Concurrently, no statistically significant differences were observed in the expression levels of Bcl-2 and TGF-β between the IR-treated groups (30.40 ± 1.68 and 59.60 ± 3.30, respectively) and the positive control groups (33.10 ± 1.80 and 66.00 ± 3.60, respectively).

Our results emphasized the maximum significant decrease in FOXP3 and CD25 expression level in the Cap@SNPs and the combined group treated with Cap@SNPs + IR (*P* < 0.01), reaching (22.80 ± 0.96 and 12.40 ± 0.65), respectively, in FOXP3 and (26.50 ± 1.11 and 19.50 ± 1.02), respectively, in CD25, when compared with FOXP3 and CD25 positive controls (36.90 ± 2.01 and 38.40 ± 2.09), respectively.

### Histopathological examination in mammary, liver and kidney tissues

**Fig. 7:**
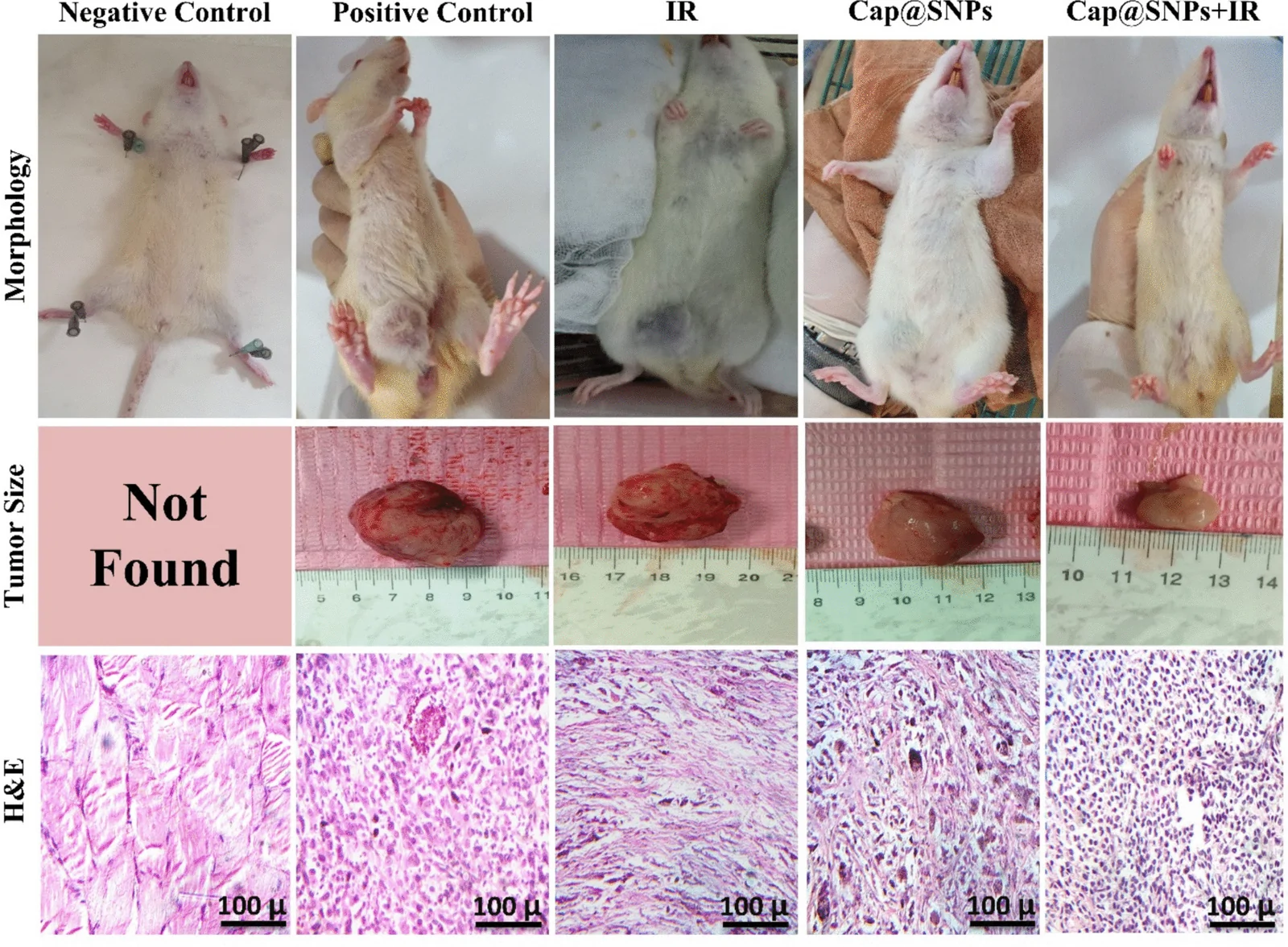
Morphology, tumor size, and histopathology statuses of breast tissues in different study groups. (*Negative control group*): Mammary tissue sections images showing healthy mammary tissue contain rounded secretory lobes within adipose tissue, each containing a compound tubular acinar gland, which discharges into ducts lined with epithelial cells. (*Positive control group*): Breast cancer cells exhibit irregular mammary tissue arrangement, increased cellularity, dysplastic changes, high proliferation index, high mitotic figures and significant stromal tissue deposition. (*IR group*): Gamma irradiation group mammary tissue exhibits tumor size reduction, tissue pattern disruption, nuclear morphology changes, fibrotic changes, increased collagen fibers and immune cell infiltration. (*Cap@SNPs group*): Cap@SNPs treated breast cancer tissue showed mild tumor size reduction, inhibition of cell proliferation, increased apoptosis, cell shrinkage and detection of new blood vessels. (*Cap@SNPs + IR group*): Breast cancer tissue treated with Cap@SNPs and radiation showed reduced tumor size, decreased cell proliferation, increased apoptosis, improved degenerative changes, reduced fibrosis and decreased inflammatory infiltrates

**Fig. 8:**
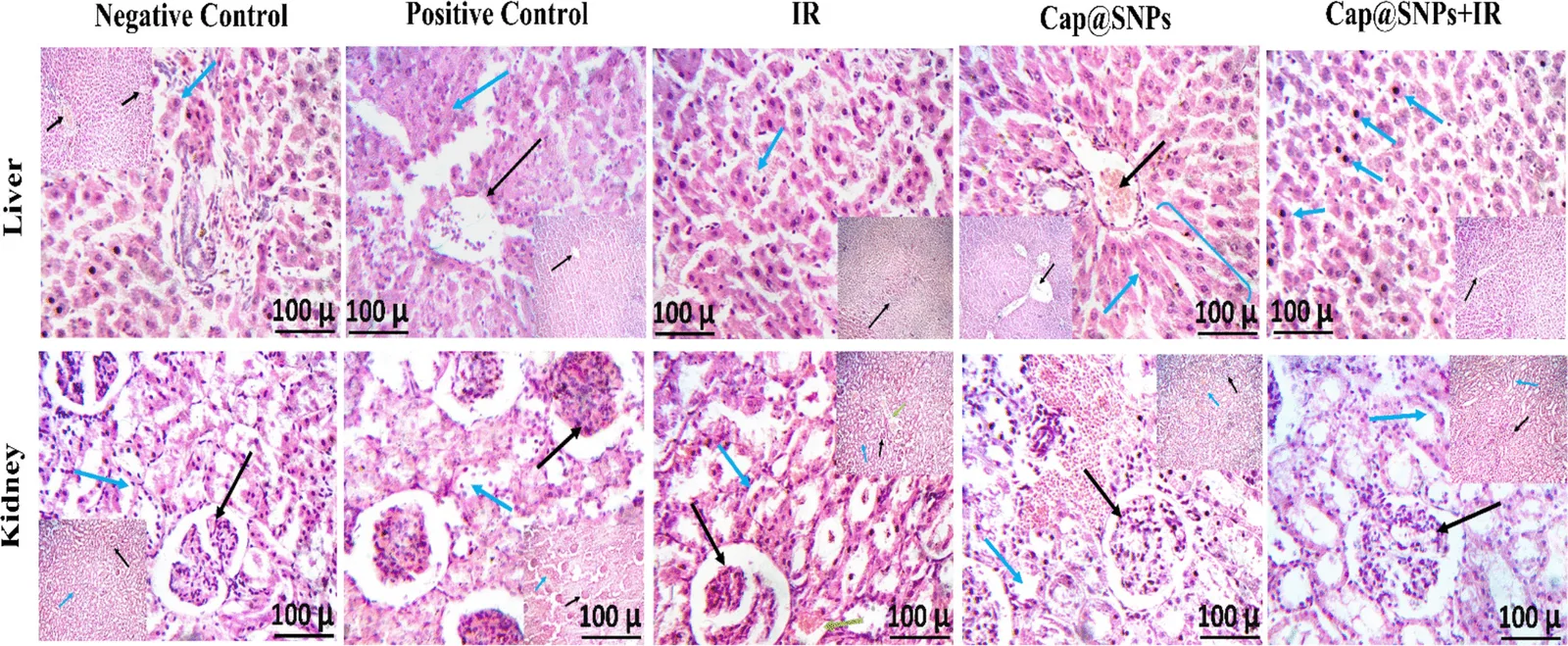
Side effects of Cap@SNPs and/or IR and histopathology statuses of liver and kidney in the different studied groups. *Liver sections images showing*: (*Negative control group*): The liver section from the control rat showed a normal histology pattern, including normal hepatic nodules and central vein. (*Positive control group*): The liver tissue of rats receiving DMBA for breast cancer induction showed signs of vascular congestion, extravasation, lymphocyte infiltration and hepatocyte ballooning. (*IR group*): The IR-treated group showed a mild grade of hepatic fibrosis, especially at the para-biliary ducts. (*Cap@SNPs group*): The Cap@SNPs group displayed mild fibrosis and extravasation, with few lymphoplasmacytic infiltrates observed in periportal and perivascular areas. (*Cap@SNPs + IR group*): Liver tissue of the Cap@SNPs & IR combined group exhibited degenerative changes, including enlarged cells, low nuclear/cytoplasmic ratio, ballooning, binucleated hepatocytes, focal lymphoplasmacytic infiltrates and grade I fibrosis. *Black arrows*: for the central vein. *Blue arrows*: for hepatocytes. *Renal sections images showing*: (*Negative control group*): The negative control group consisted of normal renal parenchyma, a thin connective tissue capsule with a glomerulus and surrounding tubules with cuboidal epithelium. (*Positive control group*): The positive control group had a thin connective tissue capsule with a glomerulus and surrounding tubules. The group also showed atrophy and degeneration of distal tubular cells, wide tubular spacing, and reticulated casts. Proximal tubules showed widening and vacuolation, while renal veins were congested. (*IR group*): The rats treated with IR showed degenerative changes in the renal parenchyma and dilated vessels compared to the negative control group, with a significant decrease in glomerular cellularity. (*Cap@SNPs group*): The Cap@SNPs group exhibited moderate renal capsule degeneration, dilated vessels, vascular endothelium degeneration, perivascular edema and fibrosis. (*Cap@SNPs + IR group*): The Cap@SNPs + IR group demonstrated renal tissue regeneration, increased glomerular cellularity and a moderate decrease in perivascular edema. *Black arrows*: for glomeruli (with their surrounding capsule, lining cells and vessels). *Blue arrows*: for renal tubules (distal and proximal). *Green arrows*: for blood vessels

Results for histopathology of mammary gland (Fig. 7) reflect that there was a mild reduction in tumor size, inhibition of cell proliferation, increased apoptosis, cell shrinkage, and detection of new blood vessels in the Cap@SNPs group. In contrast, Cap@SNPs and radiation group showed a prominent reduction in tumor size, decreased cell proliferation, increased apoptosis, improved degenerative changes, reduced fibrosis, and decreased inflammatory infiltrates when compared with the induction group that exhibits irregular mammary tissue arrangement, increased cellularity, dysplastic changes, high proliferation index, high mitotic figures and significant stromal tissue depositions. In addition, histopathology of both liver and kidney showed a significant discrimination in the construction of tissue composition in the induction group compared to the treatment groups, specifically the Cap@SNPs and combined Cap@SNPs + IR groups. The effects of Cap@SNPs and/or IR and histopathology statuses of liver and kidney in the different studied groups are shown in Fig. 8.

#### Immunohistochemical examination of HER2/neu, ER and PR in mammary tissues

Expression of human epidermal growth factor receptor 2/neu (HER2/neu), estrogen receptor (ER) and progesterone receptor (PR) was quantified using the immunoreactive score (IRS), as shown in Table 13.

**Table 13:** Immunoreactive Score (IRS) of HER2**/**neu, ER and PR on mammary tissues in different studied groups

Groups	Negative Control	Positive Control	Treated Groups		
Parameters			IR	Cap@SNPs	Cap@SNPs + IR
HER2/neu	0	8	2	2	2
ER	0	10	4	4	3
PR	1	9	4	3	1

IRS score: (0–1 = Negative), (2–3 = Mild), (4–8 = Moderate), (9–12 = Strongly Positive)

*HER2/neu* Human Epidermal Growth Factor Receptor 2**/**neu, *ER* Estrogen, *PR* Progesterone

The immunoreactive score (IRS) profile for HER2/neu in mammary tissue showed pronounced overexpression in the positive control (IRS = 8) compared with the negative control (IRS = 0). Furthermore, the treated groups with IR and Cap@SNPs, either singly or in combination, exhibited a significant decline in HER2/neu expression levels (IRS = 2, 2 and 2, respectively) compared with the positive control (IRS = 8). Regarding estrogen receptor (ER) and progesterone receptor (PR), expression levels were markedly elevated in the positive controls (IRS = 10 and 9, respectively) compared with the negative controls (IRS = 0 and 1, respectively). The treated groups with IR, Cap@SNPs, and the combination showed a significant reduction in ER expression levels (IRS = 4, 4 and 3, respectively) compared with the positive control (IRS = 10). In addition, there is a significant reduction in PR expression levels in the IR, Cap@SNPs and Cap@SNPs + IR groups (IRS = 4, 3 and 1, respectively) compared with the positive control (IRS = 9).

**Fig. 9:**
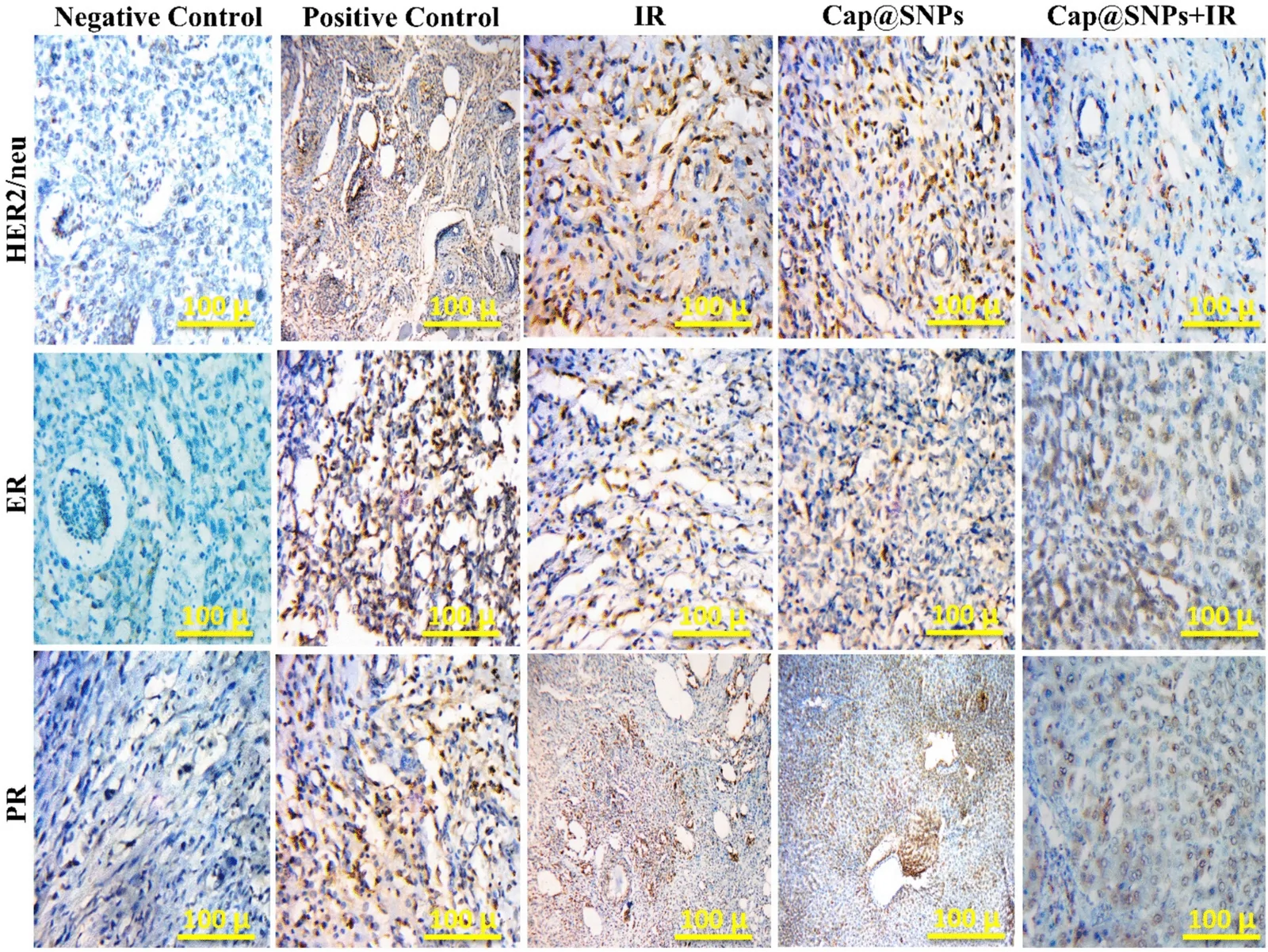
Immunohistochemical staining of BC tissue with anti-HER2**/**neu, anti-estrogen, and anti-progesterone in the studied group

The Immunohistochemical staining of mammary tissues with anti-HER2/neu, anti-estrogen and anti-progesterone in the different studied groups is shown in Fig. 9.

## Discussion

Breast cancer remains the most frequently diagnosed malignancy among women worldwide and a leading cause of cancer-related mortality. This underscores the persistent limitations of conventional therapeutic paradigms, which are often constrained by systemic toxicity, drug resistance and suboptimal tumor selectivity. In this context, a multi-targeted treatment strategy is established as a transformative therapeutic platform that integrates three complementary strategies, metronomic chemotherapy, nanotechnology-based drug delivery, and fractionated radiotherapy, into a unified and rational integrated therapeutic framework.

The era of nanotechnology has revolutionized drug delivery and targeting, changing the landscape of the pharmaceutical industry. Nanoparticles have dimensions below 100 nm, especially in drug delivery. Silica nanoparticles (MSNs) due to their ordered internal pore network with a large surface area and tunable pore sizes, facilitating high drug loading and protecting encapsulated agents from premature degradation, thereby, serving as effective drug carriers, enabling high drug-loading efficiency, facilitating surface functionalization for targeted delivery and protecting encapsulated agents from premature degradation, finally, enhancing the therapeutic index and reducing systemic toxicity. The change in administration mode facilitates precision delivery while sparing healthy tissues and mitigating the gastrointestinal adverse effects commonly associated with oral administration. Fractionated low doses of γ-radiation, when combined with chemotherapy, increase tumor susceptibility to systemic therapies and improve the potential for durable anti-cancer immune responses. The metronomic chemotherapy (MCT) strategy, using a lower long-term dose of the nano-formulation, inhibits tumor angiogenesis, modulates the immune response, and reduces systemic toxicity. Building on this optimized chemo-radio-therapeutic backbone, achieve a highly potent efficacy strategy for breast cancer treatment.

In this rational, integrated approach, the cytotoxic medication, capecitabine, was loaded onto silica nanoparticles (Cap@SNPs) and administered intraperitoneally at a low dose using a metronomic strategy, in combination with a low-fraction dose of γ-radiation, against breast cancer-bearing rats, exploits well-established mechanisms of chemo-radiotherapeutic synergy, demonstrating meaningful improvements, including enhanced DNA damage, impaired tumor repair capacity and increased radio-sensitization. Those observations and findings can be illustrated as follows.

Regarding the tumor volume, the initial tumor volume (mm^3^) across all groups was (32.8 ± 1.8) mm^3^, validating the breast cancer tumor mass induced by DMBA in the rat animal model.

Throughout the month of treatment, the untreated-induced breast cancer rats (positive control) group showed a continuous increase. From Day 5 to the end of the experiment, both capecitabine nanoformulation-treated groups (Cap@SNPs) and the combined therapy (Cap@SNPs + IR) showed highly significant (*P* < 0.01) tumor volume regression compared with the positive control. At Day 30, a significant (*P* < 0.05) reduction was observed in the γ-radiation (IR)-treated group compared with the positive control, indicating the potent effect of the newly synthesized capecitabine nano-formulation loaded onto silica nanoparticles.

After 30 days of combined treatment, the Cap@SNPs + IR-treated group showed a highly significant (*P* < 0.01) decrease in tumor volume (5.3 ± 0.5) mm^3^ compared with (86 ± 1.9) mm^3^ in the positive control group. The Cap@SNPs-treated group alone also showed a highly significant (*P* < 0.01) decrease (6 ± 0.5) mm^3^ compared with (86 ± 1.9) mm^3^ in the positive control group. At the same time, the IR-treated group showed a slight but significant (*P* < 0.05) decrease (70.2 ± 3.6) mm^3^ compared with (86 ± 1.9) mm^3^ in the positive control group. These observations indicate the potential benefits of integrated chemotherapy with a 3Gy fractionated dose of γ-radiation over 6 sessions, rather than capecitabine and IR alone, in the treatment of breast cancer.

These findings supported the antitumor effect of Cap@SNPs in a solid tumor rat model. They agreed with the findings that combination regimens of capecitabine and docetaxel in human patients with metastatic breast cancer MBC can potentially increase the time before the regrowth of the tumor and decrease tumor volume [45].

Our results revealed a significant improvement in the survival rate from 25% in the positive control group to 87.5% in the Cap@SNPs + IR combined therapy-treated group. In addition, it emphasized a 75% and 37.5% in the single-treated groups, Cap@SNPs and IR, respectively. According to the death rate, there was a decrease from 75% in the positive control to 25% in the capecitabine-treated group and 62.5% in the IR-treated group. Furthermore, showing a significant decline to 12.5% in the combined treatment (Cap@SNP + IR) group, indicating the potential benefits of integrated chemotherapy with a 3 Gy fractionated dose of the γ-radiation rather than capecitabine-treated and IR solely.

By measuring survival and tumor inhibition metrics, MST, ILS%, TIR%, and T/C%, which are used for evaluating either single/or combined-treatment therapeutic strategies' efficacy in experimental breast cancer models and according to the National Cancer Institute's (NCI) criteria, a T/C% greater than 125% and an ILS% exceeding 25% indicate significant anti-tumor activity or cytotoxicity of the drug [30] [31].

The single treatment with Cap@SNPs showed promising results, with ILS% of 40%, TIR% of 93.02% and T/C% of 140%. The combination of Cap@SNPs and gamma radiation (IR) yielded optimal results, with ILS% of 73.3%, TIR% of 93.84% and T/C% of 173.33%. thereby confirming the antitumor efficacy of Cap@SNPs singly or in conjunction with ionizing radiation. On the other hand, the application of gamma radiation alone exhibited minimal impact on the tumor area, resulting in (13.33%, 18.37% and 113.3%) for ILS%, TIR% and T/C%, respectively. The data indicate that this regimen is insufficient as a standalone antitumor treatment.

Finally, various strategies, whether implemented individually or in combination, met the specified criterion, with the exception of a single treatment involving gamma radiation. The combined therapy of Cap@SNPs + IR achieved the best results; hence, it should be approved primarily and considered an anti-tumor regimen.

Several studies have emphasized improvements in overall survival (OS) and progression-free survival (PFS) associated with capecitabine-containing regimens, as monotherapy and in combination with other chemotherapies [46]. Incorporating capecitabine into standard adjuvant chemotherapy improves survival, especially after surgery, thereby decreasing the risk of recurrence [47].

Regarding liver function (GOT, GPT) and renal function (urea, creatinine), the results showed marked elevations in the serum of positive-control rats due to BC incidence. The current combined treatment with Cap@SNPs + IR demonstrated a potential effect on enhancing liver function, with decreases of −28% and −32% in GOT and GPT, respectively, compared with the positive control. The single treatment with Cap@SNPs showed −19% and −21%, respectively, as well, gamma radiation alone recorded −13% and −15%, respectively, compared with the untreated group. The combination also demonstrated restoration of kidney function, with decreases of −44% and −59% in urea and creatinine, respectively, compared with the positive control. The single treatment with Cap@SNPs recorded −38% and −58%, respectively, whereas gamma radiation alone recorded −21% and −47%, respectively, compared with the induced BC rats. These findings indicate the potential impact of the newly synthesized nano-formulation and emphasize the synergistic effect of Cap@SNPs and gamma radiation in improving GOT and GPT enzyme activity and restoring urea and creatinine levels to normal, rather than separate treatments. Those findings agree with Simona Gurzu, 2013 and ChunLing Dai, 2015 where they found that the capecitabine treatment can increases GOT and GPT in breast cancer due to their role in those metabolic pathways, firstly, hydrolysis and conversion of capecitabine to 5'-deoxy-5-fluorocytidine (5'-DFCR) by hepatic carboxyl esterase (CE) that predominantly occur in the liver, secondly, de-amination and conversion of (5'-DFCR) to 5'-deoxy-5-fluorouridine (5'-DFUR) by cytidine deaminase (CD) that occur in liver and tumor tissues, finally, conversion of (5'-DFUR) to 5-fluorouracil (5-FU) by thymidine phosphorylase (TP), this enzyme is more active in tumor tissues than in normal tissues. These steps are crucial for the selective activation of capecitabine in cancer cells, allowing for targeted cytotoxicity while sparing most normal tissues from the toxic effects of 5-FU [48, 49].

Capecitabine has a generally favorable hepatic safety profile, with mild, transient histological changes in liver tissue, as shown in histological images of liver sections. These observations are consistent with the prior finding of not restoring liver enzymes (GOT and GPT) to normal, due to their roles in these metabolic pathways through the conversion of capecitabine to 5'-Deoxy-5-fluorocytidine (5'-DFCR) by hepatic Carboxylesterase, predominantly in the liver [50]. So, caution should be exercised when administering capecitabine to patients with mildly to moderately impaired hepatic function, as it temporarily increases liver enzymes GOT and GPT due to hepatic metabolism, but there is no need for a priori dose adjustment [51] [52].

Regarding the inflammatory markers, using the current treatment, recorded a potential effect on restoring the CRP, CA125 and AFP levels in the case of combination therapy recording decline by −35%, −61% and −72%, respectively, although the single treatment with Cap@SNPs recorded −33%, −55% and −71%, respectively, and the gamma radiation singly recorded −6%, −4% and −17% respectively, compared to the positive control.

Considering antioxidant enzyme levels (SOD and CAT), the combined therapy (Cap@SNPs + IR) resulted in 189% and 139% increases, respectively. Furthermore, the single treatment with Cap@SNPs showed an elevation of 150% and 112%, respectively. At the same time, the gamma radiation treatment alone recorded a rise of 78% and 35%, respectively.

Focusing on the lipid peroxidation marker (MDA), our results demonstrated a gradual, significant reduction in MDA levels (−25%, −60%, and −65%) in the treated groups with IR, Cap@SNPs, and Cap@SNPs + IR, respectively, compared to the positive control.

Those findings demonstrated a notable increase in both SOD and CAT levels and a significant reduction in MDA level with the integrated treatment strategy, rather than the single treatment, suggesting a potential effect of incorporating the newly synthesized nanoformulation and offering a greater impact on the multi-targeted treatment strategy than a single treatment approach. Evaluating serum oxidative stress markers may enhance the evaluation of chemotherapy efficacy and treatment response in breast cancer. Increased MDA levels signify enhanced oxidative stress, potentially facilitating cancer progression and resistance to treatment. SOD and Catalase may function as potential biomarkers for predicting treatment response or monitoring treatment efficacy in breast cancer patients receiving capecitabine chemotherapy. Capecitabine operates by generating 5-fluorouracil (5-FU) in the body, which inhibits thymidylate synthase and disrupts DNA synthesis. Oxidative stress may play a role in its therapeutic effects. Recent studies suggest that oxidative stress may influence the chemotherapy efficacy, with resistance potentially stemming from capecitabine's impact on oxidative marker outcomes [53, 54]. The association between oxidative stress and capecitabine treatment indicates the possibility of developing innovative therapeutic strategies, such as combining capecitabine with antioxidants or pro-oxidants to enhance treatment efficacy. Ebenezer et al. suggested that the combination of chemotherapeutic agents or adjunctive treatments with antioxidants like coenzyme Q10, vitamin E, and vitamin C may reduce oxidative stress by modulating antioxidant defenses or scavenging free radicals. This approach may reduce oxidative stress and related side effects caused by chemotherapy, suggesting that antioxidants could enhance chemotherapy efficacy by preventing cancer cells from entering a dormant state, thus increasing their vulnerability to treatment. The impact of chemotherapy on oxidative stress depends on the type of cancer, the chemotherapy regimen used, and the characteristics of the individual patient [55]. The findings align with those of Shan et al. who proposed that incorporating cabazitaxel into silica nanoparticles could enhance its therapeutic efficacy in breast cancer treatment and regulate oxidative stress levels. Silica nanoparticles enhance the targeted delivery of cabazitaxel to cancer cells, potentially improving effectiveness and reducing side effects by minimizing oxidative stress and tissue damage. Silica nanoparticles exhibit antioxidant properties that could mitigate oxidative stress induced by chemotherapy. This is accomplished through the modulation of antioxidant defense systems, reduction of reactive oxygen species (ROS) levels, and enhancement of antioxidant enzyme activity, including superoxide dismutase (SOD), catalase, and glutathione peroxidase, which collectively protect cells from oxidative damage. Silica nanoparticles demonstrate biocompatibility and can be engineered for the controlled release of cabazitaxel, potentially improving treatment outcomes in breast cancer therapy [56].

Regarding to the effect of the administration of the Cap@SNPs or IR, either singly or in combination, on the hematological factors, it was shown an improvement in the red blood cells (RBCs) count, the hemoglobin (Hb) concentration and hematocrit (HCT) percentage in the different treated groups, with a maximum significant elevation in the combined treated group, compared with the positive control. Many studies have reported common side effects of capecitabine as a chemotherapy agent, such as hematological toxicity, anemia, lymphopenia and neutropenia [57]. Still, our results revealed significant improvements in RBCs, HB, and HCT levels.

Relating to the white blood cells (WBCs) count, the treated group by various strategies, showing a marked increase in compared to the positive control, with a relative elevation in the single treatment by Cap@SNPs, in comparison with the other treated groups, as while chemotherapy drugs like capecitabine typically cause a decrease in white blood cell (WBC) counts (leukopenia or neutropenia), an increase in WBCs after treatment with a low dose of capecitabine in nano-formulation may be due to a biological response through bone marrow recovery, after the suppressive effect of the chemotherapy wears off, the bone marrow often enters a "rebound" phase where it rapidly produces new blood cells to replace those lost.

Focusing on the platelets (PLT), there was a highly significant decrease in platelet count in the gamma radiation (IR) treated group, compared to all other groups. This decrease is due to radiation-induced thrombocytopenia, which primarily occurs because ionizing radiation damages the bone marrow. At the same time, platelet count was elevated in both capecitabine-treated groups, whether alone or in combination with gamma radiation, compared with the positive control.

Regarding lymphocytes (LYM), a considerable decrease in lymphocyte count was observed in all treated groups, including singly Cap@SNPs, IR and combined therapy, with the greatest reduction observed in both capecitabine-treated groups. These findings demonstrate the benefits of treatment with capecitabine loaded onto SNPs (377mg/Kg), which is used in this formulation at half the conventional dose (755mg/kg).

The combination therapy of Cap@SNPs and IR recorded the highest expression of Bax by 80% (0.798 ± 0.026) and 89% elavation of Fas expression (0.892 ± 0.029), compared with the positive control. Those findings indicate that the combination is more effective at modulating Bax and Fas gene expression than either treatment alone, as fluoropyrimidines like capecitabine act as radiosensitizers by inhibiting thymidylate synthase, depleting the nucleotide pool and preventing cells from repairing DNA damage caused by radiation. In contrast, the individual treatments revealed negligible or slightly negative changes. The Cap@SNPs group showed a decrease by -10% (-0.095 ± 0.004) and IR by -3% (-0.031 ± 0.001) compared to the positive control. According to the Fas expression, it was significantly upregulated in the IR-treated group by 79% (0.786 ± 0.028), whereas the Cap@SNPs-treated group showed a slight negative decrease of -4% (-0.040 ± 0.002) compared with the positive control. For Caspase-8 gene expression, our results, compared with the positive control, showed a highly significant 51% increase in the combined-treated group (0.507 ± 0.017). In addition, gamma radiation (IR) induces intrinsic (mitochondrial) apoptosis, increasing a log fold change by 80% (0.798 ± 0.029). In the capecitabine-loaded-treated group, Caspase-8 revealed the maximum increase in log fold change by 243%, demonstrating that the capecitabine pathway strongly activates Caspase-8 (the extrinsic pathway). On the other hand, the expression of the anti-apoptotic Bcl2 gene recorded a highly significant decrease in all treated groups, with the most profound suppression observed in the IR-treated group by -238% (-2.379 ± 0.085), as gamma irradiation can induce an anti-apoptotic effect through downregulation of the Bcl-2 gene, particularly at specific low doses or chronic exposure levels, which acts as a protective “radio-adaptive” response of tumor cells, followed by the Cap@SNPs group by -168% (-1.677 ± 0.070) and the combination group by -134% (-1.344 ± 0.044). 

The cell status and its prognosis toward different regimens of treatment were evaluated through the (*Bax*/*Bcl2*) ratio. A ratio greater than 1 suggests a favorable prognosis, whereas a ratio less than 1 indicates resistance, necessitating a change in the treatment regimen to improve the outcomes [58]. The positive control group exhibits a *Bax*/*Bcl2* ratio of 0.0146, indicating high tumor growth and resistance. In contrast, the gamma radiation (IR) and capecitabine-loaded silica nanoparticles (Cap@SNPs) groups yielded ratios of 2.318 (157.7% increase) and 1.059 (71.5% increase), respectively, demonstrating a significant increase in cell death and a tendency to induce apoptosis.

Moreover, the combined therapy (Cap@SNPs + IR) provided the synergistic effect, with a *Bax*/*Bcl2* ratio of 3.673 and a 250.4% increase, reflecting the direction of mechanism of action toward apoptosis, which highlights the importance of using a multi-targeted treatment strategy, such as the one used in this study, which combined nano-improved chemotherapy with low-dose radiotherapy to avoid the resistance that can result from using a single treatment approach.

These findings agree with a study using an ER + endocrine-resistant breast cancer model, which showed improved survival, controlled tumor growth, and a lower proliferation index and higher cell death rate, demonstrating capecitabine’s ability to tilt the *Bax*/*Bcl-2* balance toward apoptosis in resistant breast cancers. It provides evidence that capecitabine-based protocols can modulate intrinsic apoptosis regulators in vivo, with lower capecitabine doses and alternating regimens being more effective at prolonging survival and controlling tumor growth than standard high-dose approaches [59]. Treatment with capecitabine alone or combined with Tarantula Cubensis Alcohol Extract (TCAE) was administered for 8 weeks in induced colorectal cancer in Wistar rats, showing that capecitabine increased *Bax* and *Caspase-3* mRNA expression while decreasing *Bcl-2*. The treatment with a combination of capecitabine and TCAE amplified these effects by increasing *Caspase-3* expression and the *Bax*/*Bcl-2* ratio [60].

Flow cytometry was used to assess the apoptosis rate in breast cancer-bearing rats after treatment with capecitabine (Cap@SNPs) and Gamma radiation (IR), singly or in combination, compared with the negative and positive controls. The distribution of cells was 10.3%, 82.8%, 7.2%, and 2.4% in the sub-G1 phase (DNA fragmentation), G1, S and G2/M phases of the negative control group, respectively. Significant deregulation of cell distribution was observed in the mammary tissue of the positive control group. Meanwhile, a considerable reduction of % change up to −65% and −57% occurred in the sub-G1 and G1 phases, respectively, noting that a significant increase in cell population of % change up to 292% and 1246% was seen in S and G2/M phases, respectively, compared to the -ve control. Regarding the effect of each of the single treatments on the cell distribution in different phases of the cell cycle, IR caused the majority of cells not to arrest at the sub-G1 and G1 phases and reached 0% and 93% respectively, this is comparable with 283% at sub-G1 and 125% at G1 phase induce arrest during the treatment with Cap@SNPs in comparison with + ve control group. By contrast, the distribution of cells in the S and G2/M phases was reduced by up to −10% and −90%, respectively, after IR treatment. Moreover, the pronounced decrease in cell population reached −74% and −95% in S and G2/M phases induced arrest after Cap@SNPs therapy compared to those of the + ve control.

Regarding the combination therapy with IR and Cap@SNPs, there was an improvement in cell distribution within the tumor tissue of the treated rats. However, there was a 400% increase in apoptotic cells (sub-G1 phase), comparable to a 113% increase in the G1 phase and an induced arrest compared with the single IR treatment. On the contrary, a significant reduction in the cell population up to −69% and −96% occurred in the S and G2/M phases, respectively, inducing arrest compared to IR treatment alone, suggesting the capability of Cap@SNPs to enhance the tumor tissue to the IR treatment by arresting the damaged cells at early stages to induce apoptosis. Annexin V-FITC staining was used to detect the tissue's apoptotic and necrotic rates. The early apoptosis rate was significantly increased in both groups treated with Cap@SNPs alone or in combination with IR, compared with the positive control group, confirming the apoptosis-inducing potential of the current nanoformulation and the potent effect of incorporating gamma radiation as a combined strategy for breast cancer treatment.

Regarding the p53 and PD-1 proteins, our results revealed a highly significant elevation in the proteins expression (*P* < 0.01), in rats treated by IR and Cap@SNPs alone or in combination, reaching (31.30 ± 1.73, 41.10 ± 1.73 and 69.10 ± 3.62), respectively in P53 and (29.40 ± 1.63, 51.50 ± 2.16 and 70.60 ± 3.70), respectively in PD-1 when compared with P53 and PD-1 in positive control (11.70 ± 0.64 and 10.20 ± 0.56), respectively. For CD4 proteins, our results revealed that there was a highly significant decrease (*P* < 0.01) in CD4 expression level in Cap@SNPs, IR and combined treated groups, reaching (25.70 ± 1.08, 29.30 ± 1.62 and 16.60 ± 0.87), respectively, compared with the positive control group (60.00 ± 3.27). There is no significant difference in CD4 expression levels between the combined treated group and the untreated negative group. On the other hand, showing a highly significant elevation (*P* < 0.01) in CD8 expression level in IR, Cap@SNPs and combined treated groups, reaching (30.90 ± 1.71, 36.20 ± 1.52 and 50.00 ± 2.62), respectively, compared with the positive control group (12.20 ± 0.66). Those findings of upregulation of P53 (tumor suppressor gene) expression agree with the previously reported study on colorectal cell lines, which showed an increase in p53 expression by the combination treatment (VPA + capecitabine/5′-DFUR + radiotherapy) in CRC cells with functional p53. This upregulation and activation of p53 are critical factors for the observed synergistic antitumor effects of the treatment [61]. Programmed cell death 1 (PDCD1), known as PD-1, is a critical immune checkpoint receptor that plays a pivotal role in modulating the immune system's response to cancer. Its primary function involves the attenuation of T-cell activity, thereby maintaining peripheral tolerance and preventing autoimmunity. However, in the context of malignancies, this regulatory mechanism is often exploited by tumor cells to evade immune surveillance. PD-1 serves as a critical regulator of T cell function, with its expression on both CD4⁺ and CD8⁺ T cells influencing the efficacy of antitumor immune responses. CD8⁺ T cells are pivotal in mediating direct cytotoxic responses against tumor cells. However, chronic antigen exposure within the tumor microenvironment can lead to sustained PD-1 expression, contributing to T cell exhaustion characterized by diminished effector functions. This exhausted phenotype impairs the ability of CD8⁺ T cells to effectively eliminate tumor cells. Notably, the presence of PD-1⁺ CD8⁺ T cells within tumors has been associated with both poor prognosis and, paradoxically, with responsiveness to PD-1 blockade therapies, depending on the context and functional state of these cells [62]. CD4⁺ T cells orchestrate immune responses by helping other immune cells, including CD8⁺ T cells and B cells. Like CD8⁺ T cells, chronic stimulation can induce PD-1 expression on CD4⁺ T cells, leading to functional impairment. This includes reduced cytokine production and proliferative capacity, which can compromise the overall antitumor immune response. Moreover, PD-1 expression on regulatory T cells (a subset of CD4⁺ T cells) can enhance their suppressive functions, further dampening antitumor immunity [63]. Our results show upregulation of PD-1 and CD8 expression and downregulation of CD4 expression, which are in agreement with work in colorectal cancer-bearing mice showing that capecitabine-based regimens can upregulate PD-1 on effector T cells within the tumor microenvironment [64].

Focusing on the level of Bcl-2 and TGF-β expression, there is a highly significant decrease in the protein expression (*P* < 0.01), in rats treated by Cap@SNPs singly or combined, reaching (23.10 ± 0.97 and 17.70 ± 0.93), respectively, for Bcl-2 and (49.30 ± 2.07 and 31.20 ± 1.63), respectively, for TGF-β, when compared with Bcl-2 and TGF-β in the positive controls (33.10 ± 1.8 and 66.00 ± 3.60), respectively. Concurrently, no statistically significant differences were observed in the expression levels of Bcl-2 and TGF-β between the IR-treated groups (30.40 ± 1.68 and 59.60 ± 3.30, respectively) and the positive control groups (33.10 ± 1.80 and 66.00 ± 3.60, respectively). Our results for TGF-β (%) showed a significant decrease in the combined group compared with the positive control group. Transforming Growth Factor Beta (TGF-β) is a multifunctional cytokine that plays a pivotal role in regulating cellular processes, including proliferation, differentiation, apoptosis, and extracellular matrix production. It is integral to embryonic development, tissue homeostasis, immune regulation, and wound healing. However, aberrant TGF-β signaling is implicated in various pathological conditions, including fibrosis, cancer, and autoimmune diseases. It acts as both a tumor suppressor and a pro-oncogenic factor, depending on the context and stage of cancer progression. In early stages, it suppresses tumors, but in advanced stages, it promotes metastasis and is associated with poor prognosis. TGF-β exhibits a dual role in tumorigenesis. In the early stages, it acts as a tumor suppressor by inhibiting cell proliferation. However, in advanced cancers, it promotes tumor progression by inducing epithelial-mesenchymal transition (EMT), enhancing cell invasion, and suppressing immune surveillance [65].

Our results emphasized the maximum significant decrease in FOXP3 and CD25 expression level in the Cap@SNPs and the combined group treated with Cap@SNPs + IR (*P* < 0.01), reaching (22.80 ± 0.96 and 12.40 ± 0.65), respectively, in FOXP3 and (26.50 ± 1.11 and 19.50 ± 1.02), respectively, in CD25, when compared with FOXP3 and CD25 positive controls (36.90 ± 2.01 and 38.40 ± 2.09), respectively. FOXP3 is a transcription factor essential for the function of regulatory T cells (Tregs), which involves the suppression of immune responses and the maintenance of self-tolerance. It is also vital for the development of strategies aimed at modulating immune responses and potentially treating immune-related diseases [66]. The results indicated a significant increase in FOXP3% in breast tissues of the positive control group compared to the normal group. Sylvain et al. proposed that FOXP3 serves as a marker for regulatory T cells (Tregs), which have the capacity to suppress the immune system and may facilitate tumor growth. The expression levels and localization (within tumor cells versus immune cells) can influence patient outcomes differently [67]. The results indicate a gradual decrease in the percentage of FOXP3 following IR treatment, with a more pronounced reduction observed in both the Cap@SNPs group and the combined group. This decrease may be attributed to the treatment's limitation on the expression of this transcription factor, thereby diminishing its effect on Tregs, which play a role in immune suppression and may affect cancer cells' response to chemotherapy [68]. CD25, or the IL-2 receptor alpha chain, is significant in breast cancer, especially regarding regulatory T cells (Tregs) and their interactions within the tumor microenvironment. CD25 is prominently expressed on regulatory T cells (Tregs), which are immune cells that inhibit the activity of other immune cells, including those involved in cancer defense. The presence of Tregs expressing elevated CD25 levels within tumors can impede the efficacy of chemotherapy by suppressing the immune system's capacity to combat cancer.

Additionally, chemotherapy may influence Tregs, potentially facilitating tumor growth and metastasis [69]. In breast cancer, elevated levels of Treg infiltration within tumors are frequently linked to adverse outcomes and may contribute to resistance to chemotherapy. The influence of chemotherapy on CD25 expression and Treg activity is multifaceted, contingent upon the specific chemotherapy agents employed, the cancer stage, and various individual patient characteristics [70]. The results of this study indicated a significant increase in CD25% in breast tissues of the positive control group compared to the normal group. Our results indicated a gradual decrease in the percentage of CD25 following treatment with IR, with a more pronounced reduction observed in both the Cap@SNPs group and the combined group. Shabnoor et al. concluded that chemotherapeutic agents may decrease the overall number of Tregs, potentially improving chemotherapy efficacy by mitigating immunosuppression [70].

While other research contradicts with the previous finding and showed that Tregs could be relatively resistant to chemotherapy compared to other immune cells, leading to an increase in their relative frequency in the presence of chemotherapy, which could offset the treatment's advantages [71]. These alterations further implicate immunoregulatory mechanisms in the overall therapeutic response, consistent with established roles of metronomic treatment in modulating the tumor microenvironment and immune dynamics in breast cancer models.

Regarding the immunoreactive score (IRS) profiles for HER2/neu, ER, and PR in mammary tissue, the positive controls showed highly significant overexpression (IRS = 8, 10 and 9) compared with the negative controls (IRS = 0, 0 and 1). The strong positive HER2/neu, ER, and PR protein overexpression validates the induction of mammary tumors via subcutaneous DMBA administration. This phenotype is driven by both hormone-dependent and growth factor–mediated oncogenic signaling, reflecting a complex tumor biology and showing enhanced sensitivity to combined treatment strategies that disrupt both DNA integrity and receptor-mediated signaling [72]. The various treatment groups, whether administered individually or in combination, exhibited a highly significant decline in HER2/neu expression levels (IRS = 2) compared with the positive control (IRS = 8). In addition, expression levels of estrogen receptor (ER) show a highly significant decrease in the treated groups with IR, Cap@SNPs and Cap@SNPs (IRS = 4, 4 and 3, respectively) compared with the positive control (IRS = 10).

Furthermore, there is a highly significant reduction in progesterone receptor (PR) expression levels in IR and Cap@SNPs, either singly or in combination (IRS = 4, 3 and 1, respectively), compared with the positive control (IRS = 9). These findings indicate that capecitabine loaded onto silica nanoparticles (Cap@SNPs) in combination with gamma radiation offers greater benefit as a multimodal therapeutic intervention for treating HER2/neu-positive, ER-positive and PR-positive breast cancer tumors. Capecitabine is more effective in ER-positive, HER2-normal metastatic breast cancer than in triple-negative disease. Hormone receptor status should guide treatment decisions and expectations for capecitabine therapy [73]. Moreover, identifying which molecular subtypes or biomarkers of BC is crucial for guiding treatment choices, predicting a suitable treatment protocol, and increasing efficacy.

Furthermore, our work paves the way for the integrated metronomic therapy protocols using a low dose of capecitabine loaded onto silica nanoparticles (Cap@SNPs) to be used for a long time as a highly effective anti-inflammatory and anti-cancer metronomic strategy in conjunction with low-dose of ionizing gamma radiation (LDRT) (< 1 Gy per fraction) shows strong effectiveness by boosting the body's immune response against tumors and making tumors more sensitive to other treatments, in addition to, enhanced sensitivity, improved absorption, toxicity reduction and maintenance of the healthy cells. Furthermore, this study suggests the potential use of the metronomic strategy in future alternating multidrug regimens. This work represents a promising shift in cancer treatment strategy, emphasizing long-term control over eradication, which could potentially improve outcomes in drug-resistant cancers. Therefore, further investigation and research on a human model are required to increase efficacy without endangering normal healthy tissues and improve quality of life.

## Conclusion

**Fig. 10:**
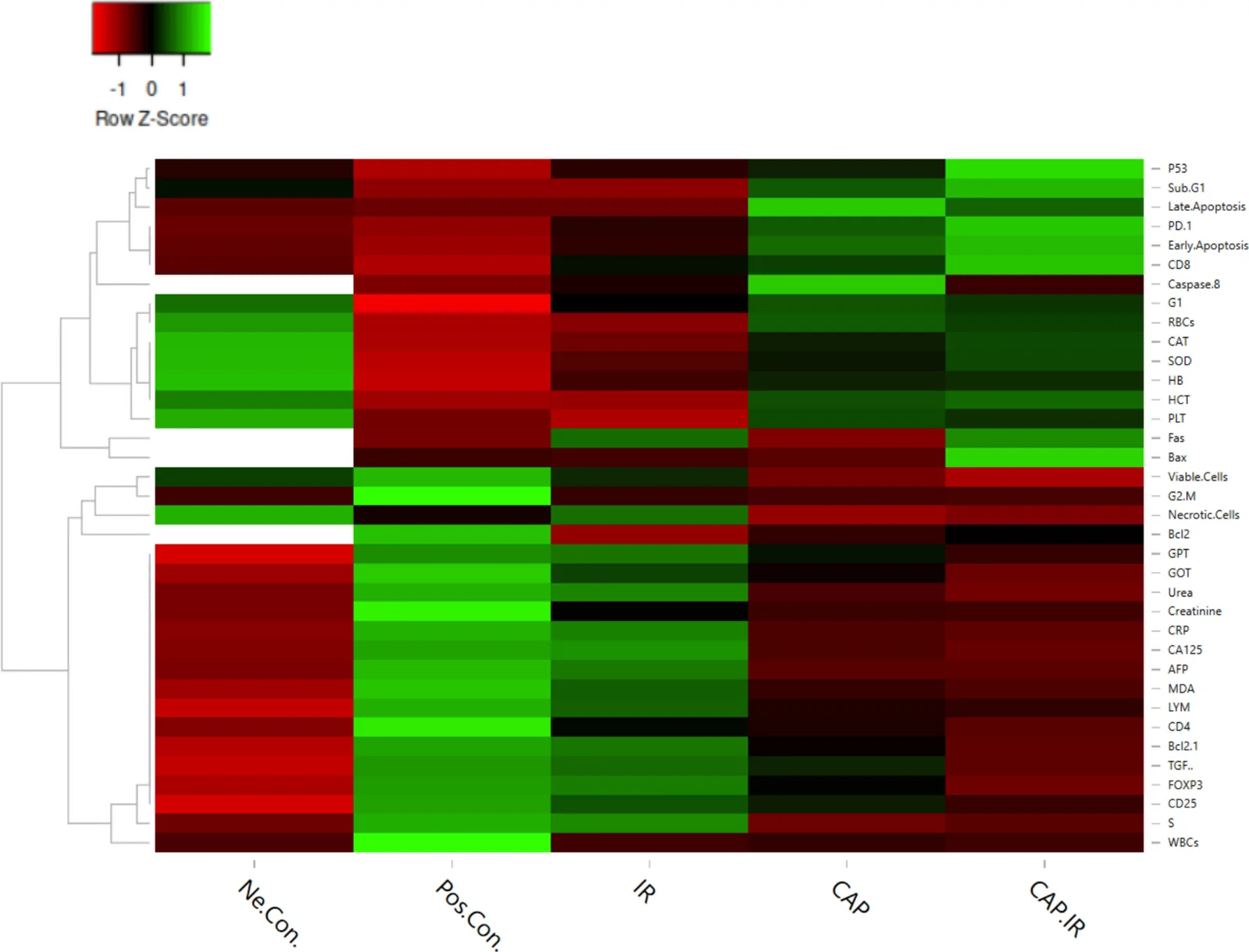
Showing the heatmap which is a powerful tool for seeing patterns in complex biological data including the hierarchically clustered heatmap for biological and hematological parameters measured across five different experimental groups: *Color Scale* (*Top Left*): It shows that: *Green* indicates a higher *Row Z-Score* (meaning a higher relative expression/level of that marker within its row) and *Red* indicates a lower *Row Z-Score* (meaning a lower relative expression/level of that marker within its row). *Dendrograms* (*Tree Structures*): These show the hierarchical clustering of markers; those that are close together on the tree tend to have similar patterns of expression across the conditions. The cancer modelling decreases the levels of these markers (e.g., P53*, Fas, Bax*, PD1, CD8, *Caspase-8*, HB, PLT, CAT and SOD), which are indicators of cancer cell resistance and proliferation. It increases the levels of the inflammation (CRP, LYM and WBCs), oxidative stress (MDA), tumor markers (AFP and CA125), liver and kidney functions (GPT, GOT, Urea and Creatinine) and anti-apoptosis and cell proliferation (*Bcl2*, FOXP3, TGF-β and CD25), which are indicators of cancer incidence and resistance. The treatment groups (low dose of gamma radiation, Capecitabine@Silica Nanoparticles and Combination): These groups show a dose-dependent or additive recovery effect, moving the parameters from the diseased state (Positive Control) back towards the healthy baseline (Negative Control). By comparing the *IR* and *Cap@SNPs* groups with the *Cap@SNPs + IR* group, it can be seen that the combined treatment exhibits a synergistic effect. While both *IR* and *Cap@SNPs* alone show some positive effects (e.g., increased apoptosis markers), the *Cap@SNPs + IR* group consistently indicates a much more pronounced shift towards a healthy profile, demonstrating that the two therapies work better together than separately. Low-dose gamma radiation acts as a *radiosensitizer*, enhancing the cytotoxic effect of capecitabine

In conclusion, the coordination of metronomic, nanoparticle-mediated capecitabine delivery with low-dose radiotherapy (LDRT) is a promising approach that can revolutionize breast cancer treatment by improving the outcomes. Collectively, this integrative strategy establishes a transformative therapeutic platform that not only amplifies treatment efficacy but also improves tolerability and long-term disease control. The observed results not only support the therapeutic efficacy of our modified drug but also warrant further mechanistic studies to elucidate its mode of action and explore its development as a targeted anticancer agent. It is first to design an integrated concurrent capecitabine dosing and low-dose γ-radiation regimen that would fill a critical knowledge gap and inform the safety, cytotoxicity, and efficacy (radio-sensitization) profiles of the newly synthesized nanoformulation. Recommend conducting additional preclinical studies of this approach and collaborating more closely with clinical investigators leading metronomic chemo-radiotherapy-based clinical trials. Furthermore, it will be important to determine which cancers are most responsive to these therapeutic approaches. There should be significant progress in oncology over the next few years. By shifting breast cancer management toward sustained, low-toxicity regimens with durable clinical benefit. Hierarchically clustered heatmap summarizing the biological and hematological parameters measured across the five experimental groups is shown in Fig. 10.

## Data Availability

The data supporting the findings of this study are available within the article.
